# Reward expectations direct learning and drive operant matching in *Drosophila*

**DOI:** 10.1073/pnas.2221415120

**Published:** 2023-09-21

**Authors:** Adithya E. Rajagopalan, Ran Darshan, Karen L. Hibbard, James E. Fitzgerald, Glenn C. Turner

**Affiliations:** ^a^Janelia Research Campus, HHMI, Ashburn, VA 20147; ^b^Solomon H. Snyder Department of Neuroscience, Johns Hopkins School of Medicine, Baltimore, MD 21205; ^c^Department of Physiology and Pharmacology, Sackler Faculty of Medicine, Sagol School of Neuroscience, The School of Physics and Astronomy, Tel Aviv University, Tel Aviv 6997801, Israel

**Keywords:** dopamine, learning-rules, decision-making, mushroom body, foraging

## Abstract

Foraging animals must use decision-making strategies that dynamically adapt to the changing availability of rewards in the environment. A wide diversity of animals do this by distributing their choices in proportion to the rewards received from each option, Herrnstein’s operant matching law. Theoretical work suggests an elegant mechanistic explanation for this ubiquitous behavior, as operant matching follows automatically from simple synaptic plasticity rules acting within behaviorally relevant neural circuits. However, no past work has mapped operant matching onto plasticity mechanisms in the brain, leaving the biological relevance of the theory unclear. Here, we discovered operant matching in *Drosophila* and showed that it requires synaptic plasticity that acts in the mushroom body and incorporates the expectation of reward. We began by developing a dynamic foraging paradigm to measure choices from individual flies as they learn to associate odor cues with probabilistic rewards. We then built a model of the fly mushroom body to explain each fly’s sequential choice behavior using a family of biologically realistic synaptic plasticity rules. As predicted by past theoretical work, we found that synaptic plasticity rules could explain fly matching behavior by incorporating stimulus expectations, reward expectations, or both. However, by optogenetically bypassing the representation of reward expectation, we abolished matching behavior and showed that the plasticity rule must specifically incorporate reward expectations. Altogether, these results reveal the first synapse-level mechanisms of operant matching and provide compelling evidence for the role of reward expectation signals in the fly brain.

## Significance

Unraveling how humans and other animals learn to make adaptive decisions is a unifying aim of neuroscience, economics, and psychology. In 1961, Richard Herrnstein formulated a long-standing empirical law that quantitatively describes many decision-making paradigms across these fields. Herrnstein’s matching law states that choices between options are divided in proportion to the rewards received, a strategy that equalizes the return on investment across options. Identifying mechanistic principles that could explain this universal behavior is of great theoretical interest. Here, we show that *Drosophila* obey Herrnstein’s matching law, and we pinpoint a plasticity rule involving the computation of reward expectations that could mechanistically explain the behavior. Our study thus provides a powerful example of how fundamental biological mechanisms can drive sophisticated economic decisions.

An animal’s survival depends on its ability to adaptively forage between multiple potentially rewarding options (1, 2). To guide these foraging decisions appropriately, animals learn associations between options and rewards (3–5). Learning these associations in natural environments is complicated by the uncertainty of rewards. Both vertebrates and invertebrates employ decision-making strategies that account for this uncertainty (6–14). A commonly observed strategy across the animal kingdom is to divide choices between options in proportion to the rewards received from each (7–15). It has been hypothesized that animals that use this operant matching strategy make use of the expectation of reward—the recency-weighted rolling average over past rewards—to learn option–reward associations (14, 15). Many studies further posit that this learning involves synaptic plasticity (16–18), and theoretical work has identified a characteristic relationship between operant matching and a specific form of expectation-based plasticity rule that incorporates the covariance between reward and neural activity (19). Despite this strong link between plasticity rules and the matching strategy, there has been no mapping of these rules onto particular synapses or plasticity mechanisms in any animal. As a result, deeply investigating these theories by manipulating and testing the nature of plasticity rules underlying operant matching has been intractable.

The fruit fly, *Drosophila melanogaster*, offers a promising system within which to address these challenges. Over the last half century, researchers have shown that flies can learn a wide variety of Pavlovian associations between cues and rewards (20–26). With the help of advances in functional and anatomical tools (27–32), they have identified the mushroom body (MB) as the neural substrate for these learning processes, including the assignment of value to sensory cues, and the underlying plasticity mechanisms have been extensively characterized (33–42). Recent theoretical work has also attempted to formalize the features of the learning rule that is mediated by these plasticity mechanisms (43–47). Despite this progress, evidence has been mixed as to whether this learning rule makes use of reward expectations (48–52), and there is a dearth of understanding about how flies learn in natural environments (but see ref. 53). Studying foraging behaviors would allow us to not only clarify these gaps in the understanding of fly learning but could also provide an insightful framework for testing the neural computations underlying decision-making strategies such as matching.

Leveraging this foraging framework in flies requires us to address several open questions. First, animals in real foraging scenarios have to be able to form associations between multiple different options and rewards, yet evidence in flies suggests that some associations are labile and easily overwritten (24). Second, choice behavior has rarely been investigated at the individual fly level (53–56), and never in the context of flies making repeated choices between two probabilistically rewarding options. It is therefore unclear whether flies can learn associations between options and probabilistic rewards. Finally, it is unknown whether they can integrate probabilistic reward events over multiple past experiences to form analog expectations. Even if such analog expectations can be formed, it is unclear whether they lead to matching behavior through covariance-based plasticity in the fly brain.

To answer these questions, we designed an olfactory two-alternative forced choice (2AFC) task for individual *Drosophila*, inspired by earlier behavior assays for flies (57–61) and foraging related 2AFC tasks in vertebrates (10–12, 14). The assay allows us to measure hundreds of sequential choices from individual flies as we vary the probability of reward associated with different odor cues. Such measurement of behavior over time allows us to distinguish between different foraging strategies, such as the matching law versus a simpler win-stay, lose-switch strategy. Importantly, our assay breaks the hard dichotomy between Pavlovian and operant conditioning. Unlike purely Pavlovian tasks (20, 24), flies in our task do not passively experience olfactory cues and rewards. Rather, the choices made by the fly dictate the odors and rewards experienced, a hallmark of operant-learning tasks. However, unlike purely operant tasks, where animals learn that specific actions lead to rewards or punishment (62, 63), flies in our task have to learn to perform stimulus-dependent actions. This relationship between stimulus, action, and reward is very similar to the dynamic foraging tasks where operant matching has been observed in other species (11–14). The dynamic foraging task structure thereby allows us to readily translate past theoretical work into the context of the *Drosophila* brain to seek a mechanistic understanding of decision-making behaviors that could apply across animals.

## Results

### Flies Learn Multiple Probabilistic Cue–Reward Associations.

**Fig. 1.**
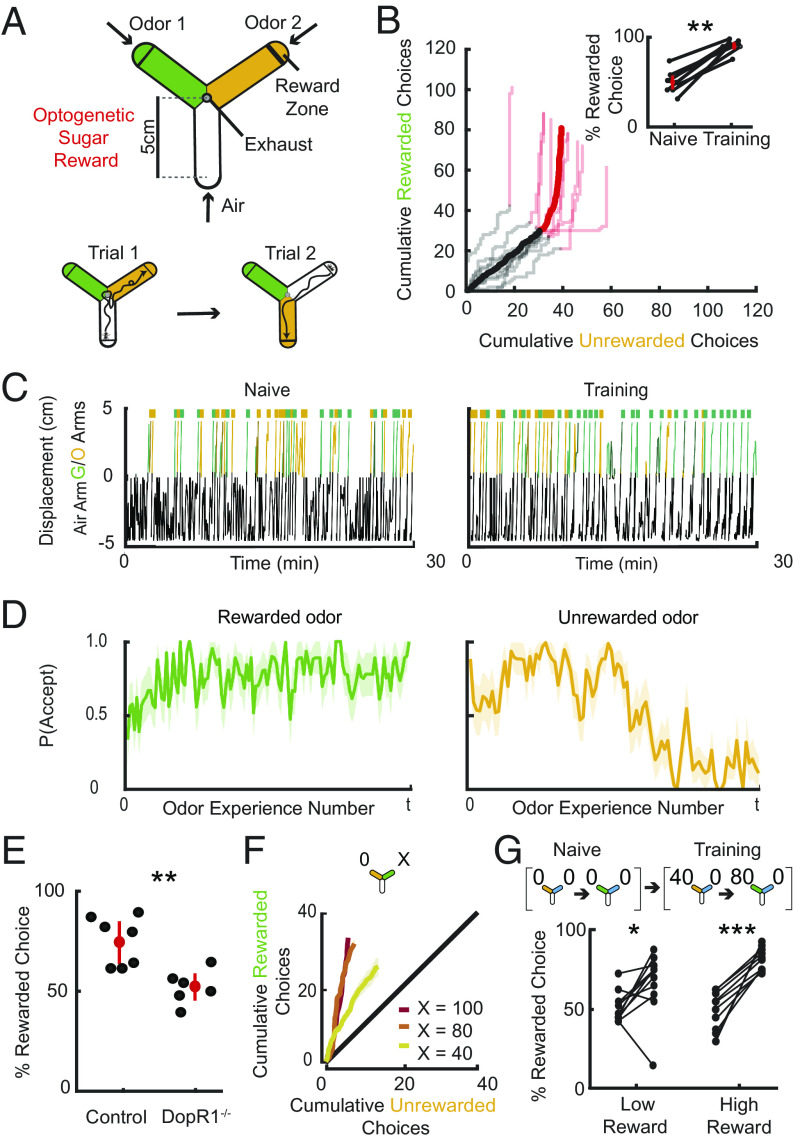
Flies learn multiple probabilistic cue–reward associations. (*A*) Schematic of Y-arena (*Top*). Air flows from tips of each arm to an outlet in the center. Reward zones are demarcated by lines. A choice is registered when a fly crosses into the reward zone of an odorized arm, triggering Gr64f sugar sensory neuron optogenetic activation with a 500-ms pulse of red light. The next trial commences as the chosen arm switches to air and the two odors (green/orange) are randomly reassigned to the other two arms (*Bottom*). (*B*) Cumulative choices made toward each option are shown (*n* = 9 flies, mean & individual flies). No rewards were available for the first 60 trials (Naive—black) and became available for the green option from the 61st trial onward (Training—red). *Inset*: Percentage rewarded choices in naive and training blocks. Flies prefer the rewarded option in the training block compared to naive (Wilcoxon signed-rank test: *P* = 0.0039, *n* = 9). (*C*) Example trajectory of a fly in the Y before (*Left*) and after (*Right*) green odor is paired with reward. Distance in air arm is represented as negative values (black), while distances in odorized arms are represented as positive values (green/orange). Choices are represented by colored rasters. At choice points the arena resets and that arm switches to air, so the fly’s position jumps to the tip of the air arm. (*D*) The probability of accept decisions are plotted as a function of time in the 100:0 protocol (*n* = 9 flies, mean—solid line, SE—shaded area). Flies show a high probability of accepting the rewarded odor (*Left*). The probability of accepting the unrewarded odor drops over time (*Right*). (*E*) Controls (*Left*) show higher percentage of choice made toward the rewarded option than DopR1 K.O. (*Right*) flies in one 100:0 block of 60 trials (mean ± SE − red point & line; individual fly scores—black; Mann–Whitney rank-sum test: *P* = 0.0022, control: *n* = 7, DopR1${}^{-/-}$: *n* = 6). (*F*) Schematic describes the reward structure of the task (*Top*). Cumulative rewarded and unrewarded choices plotted against each other, for three different protocols 100:0, 80:0, 40:0 (*Bottom*). Slope of all curves indicates that flies show a preference for the rewarded odor in all cases compared to a naive preference indicated by the black line (Mann–Whitney rank-sum test: 100:0, *P* = 4.4500 ×$10^{-8}$, *n* = 18; 80:0, *P* = 5.8927 ×$10^{-5}$, *n* = 10; 40:0, *P* = 0.0014, *n* = 10). (*G*) Schematic of the protocol for training flies with two simultaneous probabilistic cue–reward contingencies (*Top*). Two different odor choices are alternated throughout an unrewarded naive block and a reward block where options were rewarded with baiting probability of 0.4 or 0.8. Performance (percentage of choices in which the potentially rewarding option was chosen) on the low and high reward choices (*Bottom*) indicates that flies learn both associations. An increased preference for the rewarded odors over unrewarded is observed (compared to naive preference) (Mann–Whitney rank-sum test: *P* = 2.3059 ×$10^{-4}$ for high rewarding odor; *n* = 10, *P* = 0.01 for low rewarding odor, *n* = 10).

In our Y-arena, a single fly begins a trial in an arm filled with clean air and can choose between two odor cues that are randomly assigned to the other two arms (Fig. 1*A*, *Materials and Methods* and *SI Appendix*, Information 1). The fly can freely move between arms, with a choice defined as the fly crossing into the reward zone at the end of the arm (Fig. 1*A*). Once a choice is made, we provide reward by optogenetically activating sugar-sensing neurons using a Gr64f driver (64, 65). The Y-arena then resets, with the arm chosen by the fly filled with clean air and the other two arms randomly filled with the two odors. This task design permits us to precisely control reward delivery without satiating the fly and enables us to monitor the choices of a single fly over hundreds of trials.

We first established that flies learn effectively in this apparatus by reliably rewarding flies only when they chose one of the odors—what we term a 100:0 protocol. Each fly first experienced the two odors (3-octanol; OCT and 4-methylcyclohexanol; MCH) unrewarded for a block of 60 trials, and then reward delivery was activated for the following block of 60 trials. As observed previously, although individual flies exhibited different odor biases in this naive phase (54, 55, 66), those biases averaged out over the population (Fig. 1 *B*, *inset*). In this phase, flies spent a lot of time in the air arm and made variable choices, with little preference for either odor (Fig. 1 *C*, *Left*, example fly). Once reward was made available, flies rapidly shifted to choosing the rewarded odor (Fig. 1*B*). This was accompanied by a faster interval between choices (*SI Appendix*, Fig. S1*B*) and a decrease in meandering trajectories (Fig. 1*C*).

To analyze this choice behavior at a more elemental level, we adopted the common framework of considering foraging choices as a series of accept–reject decisions, where the animal decides whether or not to pursue an option (67). We defined reject decisions as when a fly enters an odorized arm but turns around and exits the arm before reaching the reward zone, while accept decisions reflect cases where the fly reaches the reward zone (*Materials and Methods*). Associating options with rewards changed the probability of accept decisions gradually over the course of a block. Acceptance probability increased for the rewarded odor and decreased for the unrewarded odor (Fig. 1*D* and *SI Appendix*, Fig. S1*E*). On average, flies were around four times more likely to reject the unrewarded odor and seven times more likely to accept the rewarded odor (*SI Appendix*, Fig. S1*D*). Interestingly, flies tended to reject odors quite close to the tip of the arm (*SI Appendix*, Fig. S1*F*), suggesting that flies might accumulate evidence over time to make and commit to their decision—an aspect of fly behavior that has previously been studied (68). These results indicate that fly choice behavior in this task can be thought of as a series of accept–reject decisions.

We found that the odor–reward associations learnt by flies in our assay were MB dependent. Learning-related plasticity in the MB circuit requires the activity of dopaminergic neurons (DANs) (24, 34, 37–41). Dopamine is sensed by odor-representing Kenyon cells (KCs) and induces synaptic plasticity between these KCs and downstream mushroom body output neurons (MBONs) (37, 39). To interfere with this plasticity, we used a tissue-specific CRISPR knock-out strategy (69) to knock out DopR1 receptors selectively in the KCs (*Materials and Methods*), which are necessary for flies to associate odors with rewards in other paradigms (41). These flies showed no detectable learning in the 100:0 protocol, compared to control animals (Fig. 1*E*). These findings establish that odor–reward associations in our behavioral assay are mediated by MB plasticity.

We then asked whether flies could link odor cues with probabilistic rewards and distinguish between different reward probabilities, a key aspect of natural foraging. Importantly, we incorporated reward baiting into our probabilistic reward tasks (12, 14). This means that rewards probabilistically become available and then persist until the reward is collected (*Materials and Methods*). Baiting is commonly used in mammalian 2AFC tasks, as it is thought to mimic the natural processes of resource depletion and replenishment over time. We began with experiments in which a single odor was rewarded with a range of baiting probabilities: 1 (100:0 task), 0.8 (80:0 task), or 0.4 (40:0 task). Flies showed a preference toward the rewarded odor in all cases compared to a naive lack of preference indicated by the black line (Fig. 1*F*). The extent of the preference varied with the probability of reward—a higher probability of reward led to a stronger preference. Interestingly, flies made faster choices when rewards were more probable (*SI Appendix*, Fig. S1*C*).

These results show that flies can learn from probabilistic rewards but do not determine whether they can store two associations simultaneously—another necessity for foraging. To test this, we designed a paradigm with a third odor, pentyl acetate (PA), included. This served as the unrewarded cue while we tested memory formation with the other two odors (Fig. 1 *G*, *Top*). Flies first made 80 unrewarded choices consisting of 40 choices between OCT and PA and 40 choices between MCH and PA. In the next 80 (Training) trials, one of OCT or MCH was assigned a high reward baiting probability (0.8) and the other a low probability (0.4). We alternated the training trials for the two different odors, to ensure that both relationships would be learnt simultaneously (*Materials and Methods*). After pairing, flies preferred both rewarded odors over PA compared to their naive preference (Fig. 1 *G*, *Bottom*). This choice preference was also reflected in their accept/reject behavior, with flies exhibiting a clear preference for accepting the high-rewarding odor (*SI Appendix*, Fig. S1 *G*, *Right*). Interestingly, in trials with the low-reward cue presented, there was an increased probability of rejecting both rewarded and unrewarded odors, as compared to naive trials (*SI Appendix*, Fig. S1 *G*, *Left*). This suggests the possibility that flies keep track of all the odor options potentially available in the environment and actually increase their rejection rate in the absence of the high-reward odor.

Overall, these experiments establish the fly as a capable animal model for studying foraging behaviors. Individual flies in the Y-arena can learn multiple odor-reward associations and can do so in the face of probabilistic reward. Importantly, these relationships are mediated by synaptic plasticity at the KC-MBON synapses in the MB. This establishes a foundation to test how these animals perform in dynamic foraging tasks and assess how they respond to reward baiting probabilities that change over time.

### Flies Follow Herrnstein’s Operant Matching Law.

**Fig. 2.**
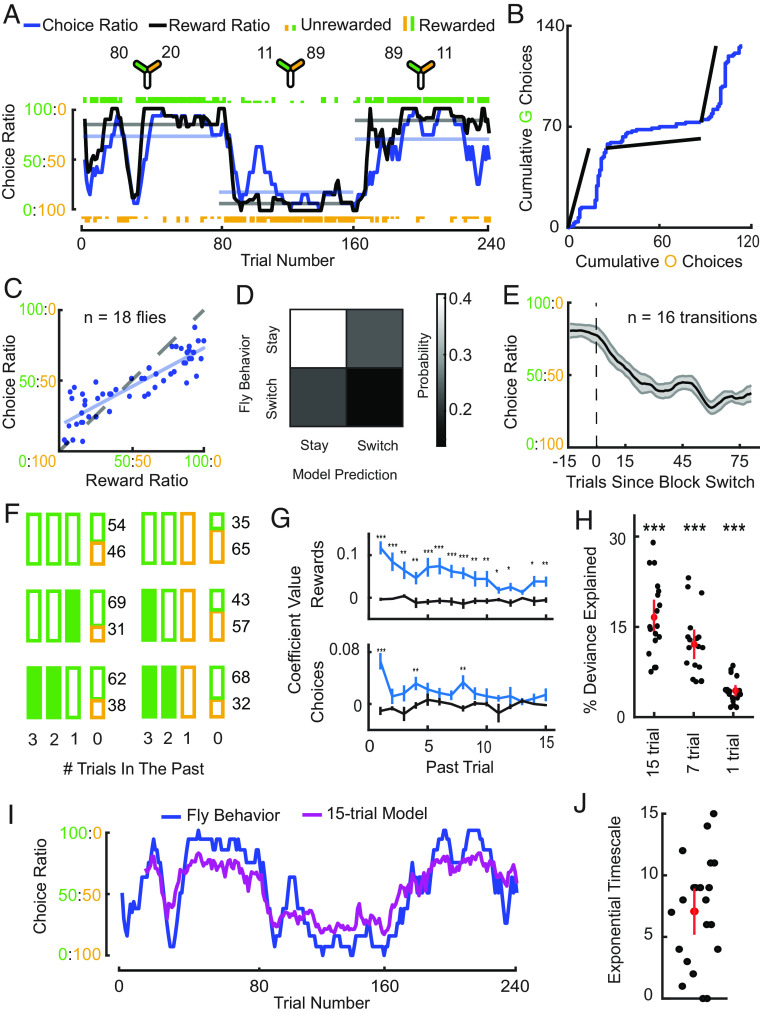
Flies follow Herrnstein’s operant matching law (*A*) Matching of instantaneous choice ratio (blue) and reward ratio (black) in an example fly. Schematics indicate the reward baiting probabilities for each odor in the three 80-trial blocks (*Top*). Individual odor choices are denoted by rasters, tall rasters—rewarded choices, short rasters—unrewarded choices. Curves show 10-trial averaged choice and reward ratio, and horizontal lines the corresponding averages over the 80-trial blocks. A description of how rewards are determined on any given trial in this task can be found in *Materials and Methods*. (*B*) Cumulative choices of the same fly. The slope of the black lines indicates the block-averaged reward ratio in each block; the blue line indicates the cumulative choices with slope representing choice ratio. The parallel slopes of the two lines indicate matching. (*C*) Block-averaged choice ratio is approximately equal to reward ratio, following the matching law with some undermatching (*n* = 54 blocks from *n* = 18 flies). (*D*) A “win–stay; lose–switch” model does not accurately capture the trial-by-trial staying and switching probabilities of flies. A 2 × 2 probability table indicating the joint probability of the action predicted by the model and the action made by the fly (*n* = 18 flies and *Materials and Methods*). (*E*) Change in instantaneous choice ratio around block changes (*n* = 16 transitions with large changes in baiting probabilities between blocks). (*F*) Analysis of choices following particular histories of experience. Choices made by flies over three consecutive past trials are represented by boxes of different colors representing odors chosen. Filled boxed indicate rewarded choices. Probabilities of choosing the green and orange odor on the current trial (0) conditional on this history are illustrated with associated values. (*G*) Coefficients from logistic regression performed on fly choice behavior to determine the influence of 15 past rewards (*Top*) and choices (*Bottom*) on a fly’s present choice (blue). These coefficients were compared to coefficients predicted on shuffled data (gray) (Wilcoxon signed-rank test: ***P< 0.001, **P< 0.01, *P< 0.05, *n* = 18 flies). (*H*) Model fit quality (percentage deviance explained) for 15-trial, 7-trial, and 1-trial logistic regression models. Null model used to calculate the quality metric is a logistic regression with 0-trial history and only bias (Wilcoxon signed-rank test comparing the null model prediction with each test model prediction; shown here as test model prediction subtracted by null model: ***P< 0.001, *n* = 18 flies). (*I*) 15-trial logistic regression fit (purple) on behavior (blue) from the example fly from panel A, plotted from the 15${}^{\;\text{th}}$ trial onward to avoid edge effects. (*J*) Exponential timescales for each fly shown in *SI Appendix*, Fig. S2, estimated from fitting the leaky integrator model (*Materials and Methods*).

Foraging tasks are cognitively complex, involving two cues paired with different baiting probabilities that change with time. This requires animals to keep track of choice and reward history and form expectations to make adaptive choices. We designed our own dynamic foraging protocol to investigate how flies behave in such a scenario. The protocol consisted of three consecutive blocks of 80 trials each. Flies made choices between two odors (OCT & MCH) that were paired with different baiting probabilities (*Materials and Methods*). These probabilities remained fixed within a block and changed across blocks (Fig. 2*A*, example).

We found that flies exhibit operant matching behavior, similar to observations in monkeys, mice, and honeybees (8, 11, 12, 14). Individual flies exhibited a strong correlation between choice ratio (defined as the ratio between the number of choices made toward option A and option B) and reward ratio (defined as the ratio between the number of rewards received upon choosing option A and option B), either calculated over entire blocks or over a short (ten-trial) window to capture short-term dynamics (Fig. 2 *A* and *B*—example fly, *SI Appendix*, Fig. S2—all 18 flies, and *Materials and Methods*). This holds true across flies, as seen in the relationship between block-averaged reward ratios and their choice ratios (Fig. 2*C*). In such a plot, the matching law predicts that all points will fall along a line with slope equal to one (the unity line). Flies appear to approximately follow the matching law with a slight amount of undermatching, signified by a slope less than one. Undermatching is commonly observed across species (11–15), and several reasons have been suggested for this tendency (13, 19) (*Discussion*).

Past work has suggested that animals form expectations of reward and use this to guide behavior in such dynamic foraging tasks (13–15, 19). When rewards are delivered probabilistically, animals can only derive an expectation of reward by tallying information over multiple trials. However, such tallying could reflect a computation beyond the capabilities of flies. We wanted to explicitly address the alternative hypothesis that flies follow a simple win–stay/lose–switch strategy (*Materials and Methods*), which would suggest that their behavior is dictated by only the most recent reward/omission experience. Simulating choice sequences using this learning rule produced output that somewhat resembled that of the fly (example in *SI Appendix*, Fig. S3*A*). However, it poorly captured the stay/switch probabilities actually observed in fly behavior data (Fig. 2*D*). In particular, switching occurred much more frequently than predicted. As further evidence that multiple past outcomes affected behavior, choices of an individual fly at block transitions showed a lag between the choice ratio curve and the updated reward ratio at transition points (Fig. 2*B*), suggesting that the fly takes a few trials to adjust its behavior. Quantifying this across multiple transitions for all flies in the task showed flies require 15 to 20 trials to reach a new steady state choice behavior following block switches (Fig. 2*E*).

It is possible that this lag could arise from averaging across multiple flies that switch at different trials after the transition. This could occur even if flies use just one past trial’s worth of information to learn about the change in reward, consistent with observations in larvae (56). To qualitatively illustrate that flies learn using multiple trials worth of past information, we first looked at the decisions made by flies following example triplets of choices and outcomes (Fig. 2*F*), inspired by recent work in mice (6). For example, following three unrewarded choices of one particular odor, flies’ next choice was roughly random (Fig. 2 *F*, *Top Left*). However, when an odor was rewarded on the most recent trial or more distant trials, choices were biased toward that option (Fig. 2 *F*, *Middle* and *Bottom Left*). In another comparison, flies’ tendency to switch back to an earlier choice (i.e., choose the green odor after an unrewarded choice of the orange odor) increased if that odor was rewarded in the recent past (Fig. 2 *F*, *Right*).

To measure the relationship between current choice and past outcomes more systematically, we used logistic regression to determine how a fly’s decisions depended on choice and reward history. Like other animals (6, 12), flies showed a small amount of habitualness by choosing options that had been recently chosen more often; regression coefficients for a short history of recent choices were significantly positive compared to coefficients fit to shuffled data (Fig. 2 *G*, *Bottom*). This approach also showed that the reward history was important for predicting choice, with many recent rewards weighted significantly (Fig. 2 *G*, *Top*). We compared regression models that predicted behavior based on different lengths of outcome histories (15, 7, and 1 trial) and found that the percentage of deviance explained over a null model with a 0-trial history was greater for models that used longer outcome histories (Fig. 2*H* and *Materials and Methods*). An example fit from a regression model with a 15-trial history is shown in Fig. 2*I*. In alignment with the results of the regression model (Fig. 2*G*), we found that when fitting a leaky integrator model(14), which assigns value to options using exponentially weighted reward histories (*SI Appendix*, Fig. S3 *B*–*E*), to the behavior of individual flies, an exponential timescale of 7 trials on average best-predicted behavior (Fig. 2*J*). Together, these results show that flies’ choices follow operant matching, with choices depending on the history of many past choices and outcomes.

### Covariance-Based Learning Is Required for Matching Behavior in a Model of the MB.

Theoretical work has placed strong, testable constraints on the nature of learning rules that could underlie operant matching. An elegant theory put forward by Loewenstein and Seung (19) proves that operant matching is the inevitable outcome of plasticity rules that modify synaptic weights according to the covariation of neural activity signaling reward and sensory input (*Materials and Methods*). Mathematically, covariance is the averaged product of two variables with at least one being subtracted by its mean. The mean is simply the average reward and/or sensory input the animal experiences—an average that can also be expressed as the animal’s expectation. Comparing the current value to its expectation ensures that weights can be adjusted up or down. Importantly, only an animal that follows operant matching would receive rewards at a rate equal to the reward expectation for both options, which leads weights to stabilize. Loewenstein and Seung mathematically formalized this intuitive link between expectation and matching and showed that when synaptic plasticity is the basis for operant matching, a covariance-based plasticity rule can account for matching.

**Fig. 3.**
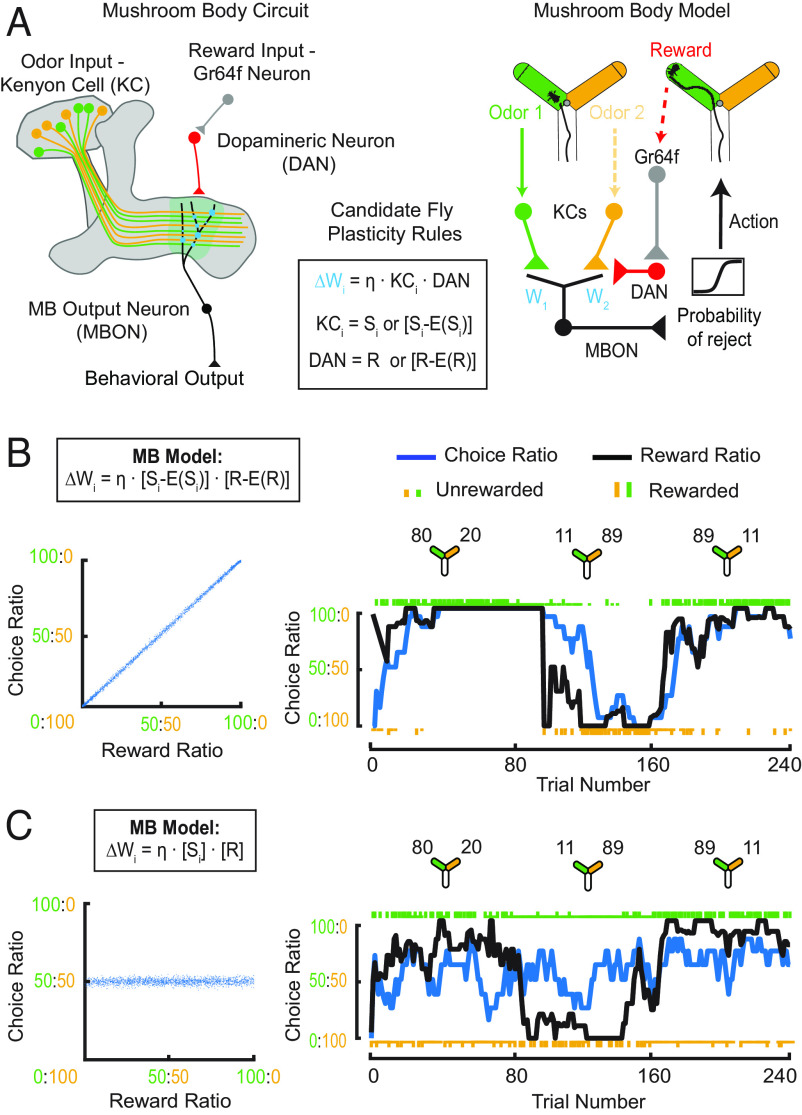
Covariance-based learning is required for matching behavior in a model of the MB (*A*) *Left*: Schematic representing the MB with all relevant neurons shown in different colors (key). *Center*: Box containing candidate reward-dependent synaptic plasticity rules at the KC-MBON synapse. *Right*: Schematic of our MB model developed by adapting Loewenstein and Seung’s model to more closely resemble the MB and the features of our olfactory task. In the modified task, agents only experience one odor at a time. Reward information is provided to this circuit via DAN activity which either represents simply reward (R) or reward minus reward expectation (R-E(R)). Weights between inputs and MBON are modified according to plasticity rules shown in *Center*, where η<0 to match the fly’s depression-based learning rule. MBON output determines probability of rejecting an odor and is passed through a sigmoidal nonlinearity to determine action. (*B*) *Left*: Block-averaged choice ratio produced by the [$S_{i}$-E($S_{i}$)] · [R-E(R)] covariance-based rule (box) plotted against reward ratio. The model exhibits matching behavior (slope is 1). *Right*: An example simulation showing the performance in a 3-block task of a model incorporating a covariance-based rule [$S_{i}$-E($S_{i}$)] · [R-E(R)]. Task reward contingencies are the same as shown for the example fly in Fig. 2*A*. (*C*) Same as (*B*), but simulated with a noncovariance learning rule. *Left*: The model produces behavior that does not show matching (slope < 1). *Right*: Performance in a 3 block task does not show matching of choice and reward ratio.

They used a simple neural circuit model to illustrate their theory, with two different sensory inputs controlling different motor outputs and a decision determined by a winner-take-all interaction between those outputs (*SI Appendix*, Fig. S4*A*). Interestingly, the structure of this model maps nicely onto the circuitry of the fly MB (Fig. 3 *A*, *Left*). Sensory inputs are represented by the KCs, each odor activating a sparse subset of the KC population (70–72). KCs synapse onto MBONs, which guide motor output by signaling the valence of an odor, i.e., its attractive/repulsive quality, rather than a specific action (22, 38, 42). KC-MBON synapses are modified by a plasticity rule that depends on the coincident activity of odor-representing KCs and release of dopamine by reward-signaling DANs (24, 34, 37–41, 43) (Fig. 3 *A*, *Center*, box). Current evidence indicates that postsynaptic activity of the MBON does not play a role in the plasticity (73), so only the sensory and reward activities need to be considered. Either or both of these terms could incorporate an expectation resulting in a covariance-based rule (Fig. 3 *A*, *Center*, box). DANs could incorporate reward expectation [(R - E(R))] by subtracting a running average of reward activity (E(R)) from the current reward-related activity (R). Similarly, KCs could incorporate sensory expectation [($S_{i}$ - E($S_{i}$))] by calculating an average sensory experience, possibly by a mechanism that involves metaplasticity and synaptic eligibility traces.

To fully adapt the theoretical framework of Loewenstein and Seung to the biological network in the MB, we had to make a few changes (Fig. 3 *A*, *Right* and *Materials and Methods*). First, odors are represented by noisy populations of KCs (70–72). We thus parameterized input representations in the model to incorporate noise and overlap of KC subsets between options. Second, in our task, flies only experience one odor at a time, so only one set of KCs is active during reward delivery. Although Loewenstein and Seung’s original theory does not account for this possibility in its proof, we extended it to this case (*Materials and Methods*). Third, plasticity between MBONs and KCs is modified by a synaptic depression rule (37, 38). We thus flipped the sign of the synaptic weight update rule. Finally, MBON activity determines whether flies accept or reject an odor rather than a winner-take-all decision mechanism (22, 38) (Fig. 3*A*). We incorporated this into our model by having MBON activity encode the probability of rejecting an odor, with higher activity representing a greater probability to reject. This MBON activity was then passed through a sigmoidal nonlinearity to determine behavioral output.

We then evaluated whether these changes affect the relationship between covariance-based rules and matching. We used this MB-aligned model to simulate behavior arising from covariance rules that incorporated stimulus–expectation, reward–expectation, or both (Fig. 3 and *SI Appendix*, Fig. S5). Consistent with the theory, all three covariance-based rules gave rise to a choice-ratio versus reward-ratio relationship that followed the matching law (Fig. 3 *B*, *Left* and *SI Appendix*, Fig. S5 *A*–*C*, *Left*). In contrast, a rule that did not incorporate either reward or stimulus expectation did not follow the matching law and instead yielded a flat slope (Fig. 3 *C*, *Left* and *SI Appendix*, Fig. S5 *D*, *Left*). For comparison, we also examined the behavior produced by the original model in a distinctly different task and observed similar results (*SI Appendix*, Fig. S4 *A*–*E*). Note that in the Loewenstein and Seung task, both options are always present when reward is delivered, which leads to a slope in between flat and unity when a noncovariance rule is used (*SI Appendix*, Fig. S4 *E*, *Left*). However, if only one option is present when an animal is rewarded, as in the fly task, synapses saturate and a noncovariance rule leads to a flat choice-ratio versus reward-ratio relationship (Fig. 3 *C*, *Left*).

To get a more refined view of model performance, we examined the trial-by-trial behavior each plasticity rule generates. Models that incorporate covariance-based plasticity rules nicely replicate the trial-by-trial behavior of flies, tracking changes in the reward contingencies across blocks, with the resulting instantaneous choice ratio biased toward the more rewarded option in each block (Fig. 3 *B*, *Right* and *SI Appendix*, Figs. S4 *B*–*D*, *Right* and 5 *A*–*C*, *Right*). On the other hand, both the MB-inspired model and Loewenstein and Seung’s model do not capture trial-by-trial behavior well when a noncovariance rule is incorporated, with choices made roughly equally to both options throughout (Fig. 3 *C*, *Right* and *SI Appendix*, Figs. S4 *E*, *Right* and S5 *D*, *Right*). This reflects the fact that when value updates only depend on sensory input and reward, plasticity is unidirectional. Consequently, synapses representing the two options will both be driven to low levels, although at slightly different rates, so that ultimately both options are chosen roughly equally. Overall, these results show that a model constrained by the network architecture of the MB more closely reproduces fly behavior when it operates according to a covariance-based plasticity rule.

### Identifying Learning Rules Underlying Dynamic Foraging in the Mushroom Body.

To test whether our theoretical prediction of a covariance-based rule is supported by the observed behavior, we developed an approach that estimated the form of the plasticity rule being used in the fly MB. Our goal was to break the plasticity rule into components that span a space of possible rules and use optimization approaches to predict trial-by-trial behavior of each individual fly to assign coefficients to each of these components. In this way, we would identify the form of the plasticity rule that best explained observed behavior and be able to conclude whether this rule was a covariance-based rule.

**Fig. 4.**
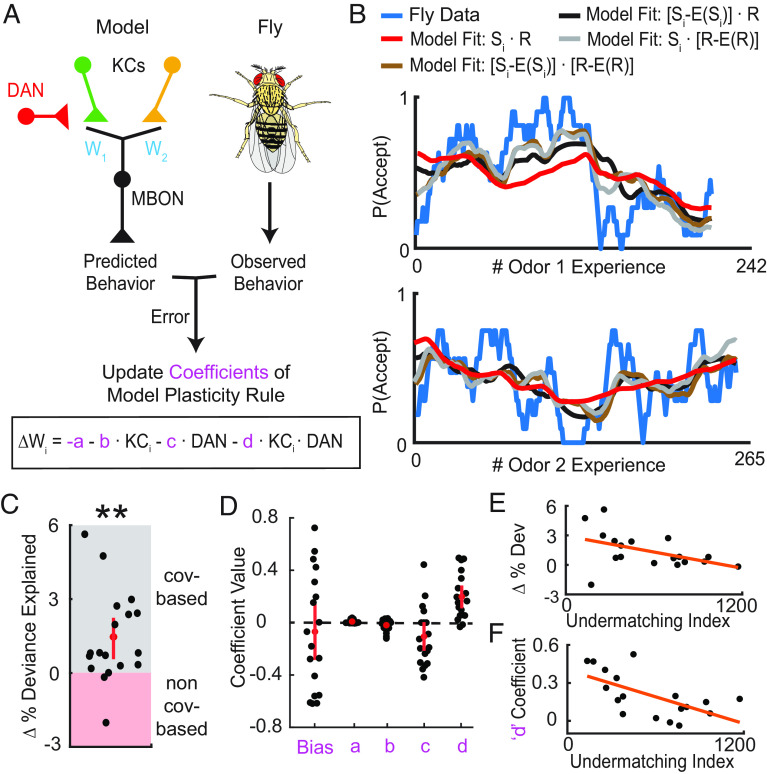
Identifying learning rules underlying dynamic foraging in the mushroom body. (*A*) Schematic detailing the logic of the MB-inspired regression model. This model was used to predict the behavior of and learning rules used by each individual fly that experienced the task described in Fig. 2. (*B*) Example fly data (blue) showing the probability of accepting odor 1 (*Top*) and odor 2 (*Bottom*) calculated over a 6-trial window as a function of the number of times the fly experienced the given odor. These data were fit using an MB-inspired regression model (*A*) that incorporates either a covariance-based rule with sensory and reward expectations (brown), just sensory expectations (black), just reward expectation (gray), or a noncovariance rule (red). (*C*) Change in percentage deviance explained, computed by subtracting the percentage deviance explained of the noncovariance-based model from a covariance-based rule that incorporates reward expectation (*n* = 18 flies). On average, fly behavior was better predicted by the covariance-based model (Wilcoxon signed-rank test: *P* = 0.0018). Individual flies that were better fit by the covariance-based model have a positive value on this plot (gray region), while flies better fit by the noncovariance-based model have a negative value (red region). (*D*) Regression coefficients assigned to each term of the plasticity rule when the MB-inspired regression model using a covariance-based rule with reward expectation was fit to the flies’ behavior. As in (*C*), the model was fit to each fly resulting in 18 different values for the coefficients. The largest coefficients were observed to have been assigned to the product term. (*E*) Change in percentage deviance explained (shown in *C*), plotted against a measure of undermatching (mean square error between instantaneous choice ratio and reward ratio lines) for each fly (*n* = 18). The best fit line of the scatter, calculated by a linear regression is shown in orange. (*F*) Coefficient value assigned to the product term (shown in *D*), plotted against a measure of undermatching for each fly (*n* = 18). The best fit line of the scatter, calculated by a linear regression is shown in orange.

We used the structure of the MB-inspired generative model (Fig. 3*A*) to build a predictive model and test how it fits the accept/rejection decisions made by the fly on each odor encounter. However, rather than utilizing a predefined plasticity rule, the predictive model used a rule composed of four terms that were candidate components of the MB learning rule (Fig. 4*A*). We then used logistic regression to assess which of these terms contributed the most when fitting fly behavioral data (*Materials and Methods*). The four terms were a constant term, a KC term reflecting sensory input, a DAN term representing reward, and finally, the product of KC and DAN activity. By definition, this product term becomes a covariance calculation when either of its elements are subtracted by their mean values, i.e., when either reward and/or sensory expectation are incorporated (Fig. 3 *A*, *Center* box). We considered four model variants, a noncovariance one that lacked any expectation term and three different covariance-based rules where either KC or DAN or both were subtracted by their expectation. At every iteration of the logistic regression, the model prediction was compared to experimentally observed fly behavior, and regression coefficients were updated. Once the fit was optimized, we evaluated which term contributed the most to the fit by examining the weights of each coefficient.

Before applying this approach to fly data, we validated it by determining whether it correctly identified the relevant term when tested with choice sequences that were simulated using a covariance-based learning rule that only incorporated reward expectation. Indeed, the fit quality was clearly better with a model that incorporated reward expectation (*SI Appendix*, Fig. S6 *A* and *B*). Moreover, the largest weights were correctly assigned to the KC-DAN product term, the term that calculates the covariance between these two elements (*SI Appendix*, Fig. S6*C*). Additionally, our simulations suggested that the extent of matching and the accuracy of learning rule fits were largely unaffected by either the degree of overlap in KC activity patterns or the timescale over which rewards were integrated (*SI Appendix*, Fig. S6 *D*–*I*). Consequently, for simplicity, we then used overlap of zero and an exponential timescale of 3.5 trials in all future analyses.

We then applied our approach directly to the behavioral data from individual flies performing the dynamic foraging task. A representative example showing fly behavior and model predictions can be seen in Fig. 4*B*. This example suggests that models with covariance-based rules may better resemble the flies’ behavior. To quantitatively compare fit quality of the different models, we calculated the percentage deviance explained for every individual fly. This metric showed that regressions that utilized rules with sensory expectation, reward expectation, or both were objectively better fits for fly behavior (Fig. 4*C* and *SI Appendix*, Fig. S7*A*).

Overall, we found that learning rules that incorporated either sensory or reward expectation both yielded better fits to fly behavior than noncovariance rules. To distinguish between these different expectation-based learning rules, we examined which regression coefficients had the biggest weights. When we fit a rule with only reward expectation, the regression assigned the KC-DAN product, i.e., the covariance term, with the largest weight (Fig. 4*D* and *SI Appendix*, Fig. S7*B*). On the other hand, fitting using either of the two covariance rules that incorporated sensory expectations yielded large coefficients for the noncovariance terms containing either KC or DAN activity alone (*SI Appendix*, Fig. S7*B*). We observed a similar result when we fit simulated data from an agent using a reward expectation–based learning rule (*SI Appendix*, Fig. S7*C*). Nevertheless, when the behavior was simulated using the same sensory expectation rule, the covariance term was given the most weight (*SI Appendix*, Fig. S7*E*). These results suggest that flies use a covariance rule based on reward expectations to guide their behavior.

Interestingly, we found that in some flies, the simple expectation-free noncovariance rule was a better fit. One possible explanation for this result is that these flies showed operant matching to a lesser extent. We thus quantified matching by calculating the mean squared error between instantaneous choice and reward ratios and found that different strengths of matching across flies were correlated with how well an expectation-free plasticity rule fit the behavior data (*Materials and Methods*). Flies that were better fit by the expectation-free rule tended to show more undermatching, in line with our predictions (Fig. 4*E*). Consistent with this, the weight of the covariance term coefficient was greater in flies that exhibited stronger matching behavior (Fig. 4*F*). To examine whether some flies were better fit by a noncovariance rule because our approach might inaccurately assign weights to a combination of correlated terms in the learning rule, we examined the correlations between pairs of coefficients. However, we found no consistent statistical relationship (*SI Appendix*, Fig. S7*D* and *Materials and Methods*). Overall, this general approach allowed us to estimate the learning rule the fly uses directly from behavioral data, providing clear evidence that a reward-expectation-based covariance rule is important in the MB.

### Behavioral Evidence of Reward Expectation in DANs.

We next wanted to experimentally verify that a reward-expectation-based covariance rule in particular guided learning and choice behavior in the fly MB. The mathematical differences between the three different covariance rules suggested a way forward (*Materials and Methods*). In particular, the rules differ in which terms—sensory input or reward—incorporate an expectation. Thus, to distinguish between the possible different covariance-based rules in the MB, we designed an experiment to manipulate the calculation of reward expectation using genetic tools that override the natural activity of the DANs. Specifically, we provided reward via optogenetic activation of the reward-related protocerebral anterior medial (PAM) DANs. This would bypass any upstream computation of reward expectation and simply provide a consistent reward signal on every trial. Such a manipulation would change the learning rule from a covariance-based rule to a noncovariance rule if the following conditions were met: i) the animal’s learning rule depended on the product of DAN and KC activities; ii) DAN activity incorporated reward expectation; and iii) KC activity did not incorporate sensory expectation. This would in turn result in modified behavior. For this test, we initially focused on a task consisting of two blocks (naive and training) of 60 trials each, with a reward ratio of either 100:0 (one odor has a baiting probability of 100% and the other is never rewarded) or 80:20 (one odor has a baiting probability of 80% and the other 20%) in the second block (*Materials and Methods*).

**Fig. 5.**
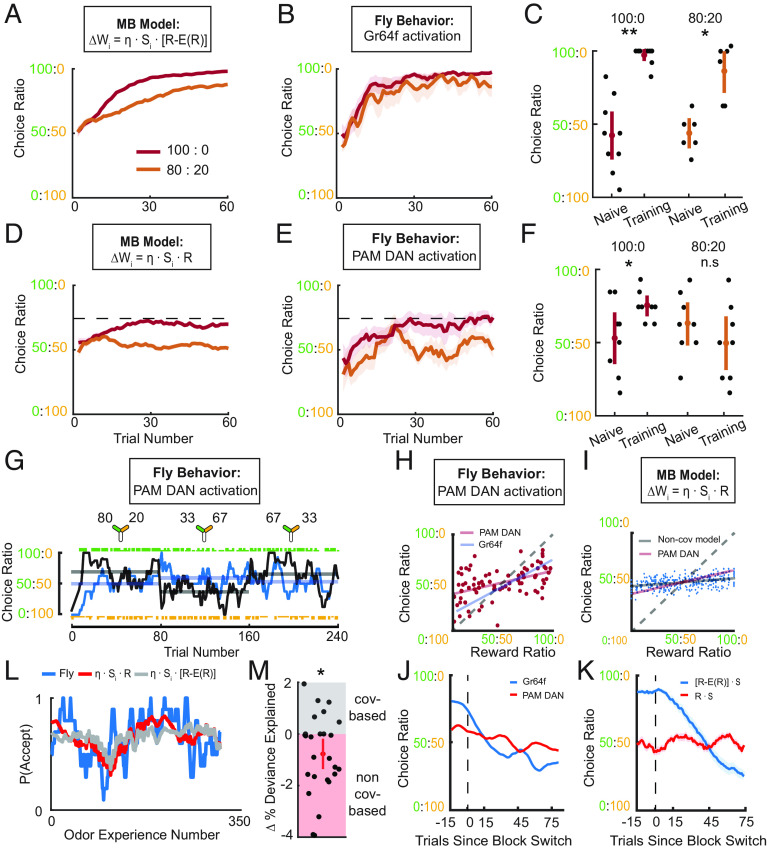
Behavioral evidence of reward expectation in DANs. (*A*) Instantaneous choice ratio over trial number, for a simulated agent using a covariance-based rule with reward expectation in 80:20 (orange) and 100:0 (red) tasks. (*B*) As (*A*), except it shows fly behavior when providing sugar sensory optogenetic reward instead of simulation (100:0, *n* = 8 flies; 80:20, *n* = 6). (*C*) Average choice ratios of individual flies from (*B*) showing significant learning in both 100:0 and 80:20 protocols (Wilcoxon signed-rank test: 100:0, *P* = 0.0039; 80:20, *P* = 0.0312). (*D*) As (*A*), except for an agent using a noncovariance rule. (*E*) As (*B*), except reward provided via the PAM DANs using R58E02-Gal4 to drive UAS-CSChrimson (*n* = 8 flies in both 80:20 and 100:0). Dashed line in (*D*) and (*E*) indicates the maximum possible performance of agent in *D* in the 100:0 protocol. (*F*) Average choice ratios of individual flies from (*E*). Flies showed a significant preference toward the rewarded odor in 100:0 but not 80:20 (100:0, *P* = 0.0391; 80:20, *P* = 0.1875). (*G*) The instantaneous choice ratio of an example fly receiving DAN optogenetic reward performing the dynamic foraging protocol plotted against trial number as in Fig. 2*A*. (*H*) Block-averaged choice ratios against reward ratios for flies with DAN reward (*n* = 26 flies, 3 blocks each). Best fit lines : red—DAN reward, blue—Gr64f sugar sensory reward (Fig. 3*C*). (*I*) Block-averaged choice ratios against reward ratios (*n* = 50) from data simulated using a noncovariance-based rule. Best fit lines : black—simulated data, red—DAN reward. (*J*) Instantaneous choice ratio around block changes. Flies trained with Gr64f activation in blue, DAN activation in red. (*K*) As (*J*) but with simulated agents using either a covariance-based rule in blue or noncovariance rule in red. (*L*) Example fly data showing probability of accepting odors against experience number (blue) with DANs activated as reward. Fit using a model (Fig. 4) that incorporates either a covariance-based rule (gray) or a noncovariance rule (red). (*M*) Change in percentage deviance explained, computed by subtracting the percentage deviance explained of the noncovariance-based model from a covariance-based rule, plotted for each fly (*n* = 26). On average, fly behavior was better predicted by the noncovariance-based model (Wilcoxon signed-rank test: *P* = 0.0164).

We first predicted how behavior in these protocols would differ between covariance-based and noncovariance rules using simulations. As expected, covariance-based models learnt to choose the more rewarded option more often, with choice ratios reflecting reward ratios (Fig. 5*A* and *SI Appendix*, Fig. S8 *A* and *B*). The behavior of the model with any covariance-based rule was similar to the fly behavior when it was rewarded using the sugar neurons (Fig. 5 *B* and *C*). On the other hand, noncovariance rules led to preferences saturated around 75% in 100:0 and 50% with the 80:20 reward ratio (Fig. 5*D*). These theoretically predicted preferences very closely match our observations of fly behavior in the DAN activation experiments (Fig. 5 *E* and *F*). We observed low plateau performance in both tasks (Fig. 5 *E* and *F*), with values strikingly similar to that predicted by the noncovariance rule (Fig. 5*D*).

One potential concern with these experiments is that differences in the efficacy of optogenetic activation of the DANs deep in the central brain versus the peripherally located Gr64f neurons could contribute to these behavioral differences. However, when flies were instead made to choose between reward-associated or unrewarded odors in a circular arena previously used to assess learning in flies (24), we found that both PAM DAN and Gr64f sugar neuron activation drove similar learning (*SI Appendix*, Fig. S9 *A*–*D*). Since the LED intensity in the circular arena (2.3 mW/cm${}^{2}$) was closely matched to that in the Y-arena (1.9 mW/cm${}^{2}$), differences in optogenetic efficacy cannot explain the range of behavioral patterns seen in the circular and Y arenas. All data are consistent with the interpretation that PAM activation bypasses the computation of reward expectation and converts a covariance rule into a noncovariance rule. In particular, learning via the noncovariance plasticity rule only modifies weights from Kenyon cells that respond to the rewarded odor, which increases the acceptance probability of the rewarded odor without changing behavior to the unrewarded odor. According to this model, performance saturates in the Y-arena because the fly repeatedly encounters the unrewarded odor by chance, and their initial tendencies for accepting the odor option never change; performance does not saturate in the circular arena because a fly that has learned to accept the rewarded odor will stop exploring and cease to encounter the unrewarded option.

We next examined how bypassing reward expectation affects matching behavior. When tested with the same three-block matching design as earlier, but now providing a consistent reward signal via direct DAN stimulation, flies exhibited strongly diminished matching behavior (Fig. 5 *G* and *H*). The slope of the choice-ratio versus reward-ratio plot was lower than that observed with Gr64f-driven reward and approached the flat line predicted by simulations of behavior with a noncovariance based learning rule (Fig. 5*I*). The instantaneous choice ratio and reward ratio of an example fly (Fig. 5*G*) suggested that this flattening arises because choice ratios are never strongly biased to either odor. This is again explained by the unidirectional noncovariance rule. In agreement with this, changes in choice ratio at block transitions were much flatter with DAN reward than with Gr64f, as expected by the differences between the covariance-based and noncovariance models (Fig. 5 *J* and *K*). To quantitatively evaluate whether providing reward with DAN activation changed the learning rule from covariance-based to a noncovariance rule, we fit our MB-inspired regression models (Fig. 4*A*) to fly data produced with DAN reward. We found that the noncovariance rule was the better fit (Fig. 5 *L* and *M*). We find through these experiments that bypassing the computation of reward expectation changes fly choices from resembling behavior produced by a covariance-based learning rule to behavior expected from a noncovariance rule. In particular, the results suggest that this covariance-based rule is located in the fly MB and incorporates reward expectation but not sensory expectation.

Altogether, our results support the theory that covariance-based learning rules that incorporate reward expectation underlie operant matching in flies. It suggests that a reward expectation signal is calculated in the DANs of the fly MB and provides the first mapping of learning rules underlying operant matching onto plasticity mechanisms at specific synapses.

## Discussion

The foraging strategies used by animals play a key role in their survival. Operant matching is one simple and ubiquitous behavioral strategy, utilized in dynamically changing and probabilistic environments. Despite the ubiquity of this strategy and its strong theoretical foundation, little is known about the underlying biological mechanisms. We leveraged the growing body of knowledge regarding learning in the fruit fly, and the plethora of available anatomical tools, to identify these mechanisms. We developed a foraging task that allowed us to monitor choices of individual fruit flies and showed, for the first time, that flies follow Herrnstein’s operant matching law. Combining experimental results with computational modeling, we found that this behavior requires synaptic plasticity and uses a rule that incorporates expectation of reward via the rewarding PAM DANs. Our results provide the first mapping of the learning rule underlying operant matching onto the plasticity of specific synapses—the KC-MBON synapses in the MB.

### Does the Ubiquity of Operant Matching Imply a Common Mechanistic Framework?.

When choosing between options that predict reward with different probabilities, mammals, birds, and insects all follow Herrnstein’s matching law (8, 9, 11–15). This is clear at the global, trial-averaged level, where choice ratios are roughly equal to reward ratios, but is also true at the trial-by-trial level (Fig. 2*A*). In fact, we found that individual choices made by flies depended on choice and reward information received over multiple past trials (Fig. 2 *G* and *H*). This is in agreement with what has been observed in mice and monkeys (12, 15) and suggests that these animals all make use of similar kinds of information to guide their behavior. Flies also show an increase in speed of choice when rewarded, another common signature of learnt behavior in mice and monkeys (11, 12) (*SI Appendix*, Fig. S1 *B* and *C*).

It is unclear whether these behavioral similarities result from underlying mechanisms that are shared across species. At its surface, mechanistic similarities seem likely. For example, neural signals that subtract reward expectation from reward—a key component of the plasticity rules underlying matching shown here—can be found in the form of a reward prediction error in many different animals (74, 75). Nevertheless, such a signal on its own is not sufficient to produce matching; it needs to be incorporated into a covariance-based plasticity rule in a behaviorally relevant circuit. On the other hand, while learning values of options via synaptic plasticity is the traditional mechanistic framework thought to underlie such foraging decisions (16, 17), recent work has found signatures of graded neural responses proportional to value during inter-trial-intervals, suggesting a persistent-activity-based mechanism for foraging decisions that may not require synaptic plasticity (12, 76). Associated modeling efforts suggest matching can arise from models that don’t incorporate synaptic learning (19, 77).

While both synaptic plasticity and nonplasticity mechanisms can explain the observed behaviors, each makes different testable assumptions about the underlying neural architecture (18) and the effect of changing environmental conditions on the behavior. For example, if one eliminated reward baiting in our experiment, a circuit using a covariance-based plasticity rule would still give rise to behavior that follows Herrnstein’s matching law. In this case, following such a law would lead the animal to always choose the option with higher reward probability. On the other hand, if matching behavior was produced using a different mechanism, the lack of reward-baiting might give rise to different strategies, such as the probability matching strategy commonly observed in mice under these conditions (6). Experiments to identify which mechanisms are used by different brains, and theoretical work to understand why, would therefore provide important insight into circuit function and the neural basis of operant matching.

### Beyond Covariance-Based Synaptic Plasticity.

Our behavioral evidence suggests that synaptic plasticity in the mushroom body depends on reward expectations through a simple covariance-based plasticity rule. We identified this plasticity rule by the process of elimination. First, we narrowed our focus to the three minimal covariance-based plasticity rules inspired by the architecture of the MB. Importantly, Loewenstein and Seung showed that these rules produce matching. We then showed that only one of the three rules also explains the results of the DAN-activation experiment. It’s important to recognize that more complex plasticity rules may be consistent with our data and necessary to explain future mechanistic and behavioral data. For instance, the plasticity rule could be augmented by adding any term that averages to zero in the matching task. The plasticity rule could also be changed to involve a nonlinear function of the current synaptic weight, presynaptic KC activity, and postsynaptic MBON activity. The fundamental requirement of Loewenstein and Seung’s theory is merely that the plasticity rule ultimately drives the covariance between neural activity and reward to zero.

Loewenstein and Seung’s theory provides an impressively general link between operant matching and covariance-based plasticity, but it does make several assumptions that may be violated in the fly. For instance, the theory assumes that plasticity only occurs when the animal makes a choice, with weights fixed between decisions (*Materials and Methods*). In our current paradigm, this means that no plasticity occurs when the fly rejects an odor or otherwise explores and navigates through its environment. In contrast, DANs encode a variety of motor variables and are not locked to choice or reward (39, 78). These motor-related DAN signals would presumably modify synaptic connections in the MB, and such off-task plasticity could generate important variability in synaptic weights and choice behavior. Interestingly, recent work has also found that these same DANs do not have a consistent effect on action-reward learning in a purely operant task (63). This suggests that motor-related DAN signals are not the substrate for operant learning, and MB plasticity may specifically act to link sensory cues to rewarding actions. Further, the theory assumes that neural activity and reward are conditionally independent given choice. The MB represents reward via DAN activity, so this assumption could be violated if KC and DAN activity have correlated variability across trials that is not related to choice. Such correlations are feasible given indirect connections from KCs to DANs and the complexity of DAN activity (31, 32, 78). Finally, the theory pertains to tasks where the animal decides between two options. Some animals have also been found to exhibit operant matching behavior when choosing between three or more options (8, 79). In this setting, operant matching still implies that the covariance between neural activity and reward vanishes, so there is hope that covariance-based plasticity rules would generate matching. However, other behavioral strategies can also lead to vanishing covariance (*SI Appendix*). It would be interesting to investigate whether modified learning rules can more reliably produce matching in naturalistic foraging scenarios or multioption choice tasks.

### Plasticity in Multiple MB Compartments Could Explain Deviations from Matching.

One complication to the framework of expectation-based learning rules and matching is that flies, like several other animals, don’t perfectly follow the matching law; rather they undermatch. Two hypotheses have been proposed to account for this deviation. The first proposes that animals that undermatch make use of a learning rule that deviates from a strictly covariance-based rule (19). Synaptic saturation and representation of motor variables in DAN response, as discussed in the previous section, offer particularly simple possibilities. Another important possibility for how this could occur is to have plasticity at multiple sites contributing to the overall learning, with different plasticity rules at each site. Indeed, the MB is divided into multiple compartments that contribute to behavior but exhibit important differences in learning (22, 24). It is possible that some compartments make use of reward expectation in a covariance-based learning rule, while others do not. Alternatively, undermatching can also result if reward expectations are estimated over long timescales (13), even if all compartments made use of a covariance-based rule. This idea suggests that in a dynamic environment where baiting probabilities change quickly, the memory of past experiences acts as a bias that prevents the animal from correctly estimating the present cue–reward relationships. This is possible in the MB, as different compartments form and decay over different time scales (24). Whether either or both of these hypotheses explain undermatching in flies can be studied in future experiments by manipulating different compartments of the MB circuitry and analyzing the effect of such a manipulation on undermatching. Relatedly, it would be interesting to check whether animals could adapt the timescales used to estimate reward expectations to the dynamics of the behavior task.

### An Approach for Inferring Learning Rules from Behavior.

Here, we introduced a statistical method that uses logistic regression to infer learning rules from behavioral data. While we specifically applied our approach to infer learning rules for the fly mushroom body, the inference of learning rules is of importance to many areas of neuroscience (80–82). In fact, this method could be similarly applied to model other learnt behaviors in the fly and other animals. In the current work, we considered learning rules that only depended on the current sensory stimulus (KC response) and reward (DAN response), but our methodology would also generalize to the inference of learning rules that incorporated a longer time-scale history of sensory input and reward. For example, the framework would be able to estimate rules that incorporated the weighted average of recent sensory experience.

However, it’s important to realize that the logistic regression formalization would break down entirely for learning rules that depend on the magnitudes of synaptic weights or postsynaptic activity. Such terms would induce different nonlinear dependencies between the choice sequence and learning rule parameters, preventing us from converting these choice and reward histories into regression inputs related to each component of the learning rule (*Materials and Methods*). Our approach was appropriate here because the plasticity rule in the mushroom body is not thought to involve these terms. However, many biological learning rules do depend on postsynaptic activity and current synaptic weights, and future work should explore more flexible methodologies from modern machine learning to develop generally applicable approaches (82).

### Circuit Mechanisms for Matching and Reward Expectation in Drosophila.

We have shown that operant matching is mediated by synaptic plasticity in the fly mushroom body and involves the calculation of a reward expectation. However, the mechanisms underlying this calculation remain unclear.

The proposed mechanism underlying the calculation of reward prediction error (RPE) in mammals provides a hint at one option (74). Here, dopaminergic neurons implicitly represent expectation by calculating the difference between the received reward and the reward expectation. This has been found to involve the summation of positive “reward” inputs and negative GABA-ergic “expected reward” inputs to the dopaminergic neurons (83). MB DANs could represent reward expectation in a similar way. In fact, the recently released hemibrain connectome (32) has found many direct and indirect feedback connections from MBONs to DANs that theoretical work has shown could support such a computation (43, 47). In the MB circuit, MBON activity is related to the expectation of reward associated with a given odor (22, 38, 42). An inhibitory feedback loop, via GABA-ergic interneuron(s) for example, could potentially carry reward expectation–related information from MBONs to DANs. The negative expected reward signal from this interneuron could be combined in the DANs with a positive reward signal from sensory neurons, allowing DAN activity to represent the type of reward expectation signal needed by a covariance-based rule.

It is important to note that such a mechanism would have a major difference from mammalian RPEs. Since MBON activity is linked to the presence of odor, the reward expectation signal would vary across stimuli and only be present when the stimulus was too. Thus, this signal would not have the temporal features of mammalian RPEs. This difference in temporal structure of the reward expectation signal could explain the mixed observations from past studies aimed at identifying reward expectation in flies. For instance, a study that used temporally distinct cues and reinforcements suggested that DANs do not incorporate reward expectation (49), while studies that used temporally overlapping cues and reinforcements did find signatures of reward expectation (48, 52), albeit with different temporal properties than the typical mammalian RPE.

It’s also possible that reward expectations are incorporated into mushroom body plasticity by adjusting the levels of reward and punishment needed to achieve a given dopamine signal. In this scheme, reward-related dopamine neurons could represent how much a reward exceeds expectations, and punishment-related dopamine neurons could respond when expectations are not met. This is reminiscent of the idea from Felsenberg et al. that interactions between reward and punishment-related compartments in the MB guide bidirectional learning (26, 45, 46, 51). However, here we extend the idea by proposing that reward would not only modify KC-MBON synapses but also modulate the baseline dopamine release or firing threshold of reward-related dopaminergic neurons. Similarly, upon missing an expected reward, learning would do the same for MBONs and DANs in punishment-related compartments. The resulting behavior would depend on the balance between the activity of both reward and punishment compartments; if the reward and punishment baselines were updated correctly, such a mechanism could produce a covariance-based rule and support operant matching. This mechanism would also tie into the notion that phasic dopamine release (i.e., the difference of dopamine from its baseline level) mediates the RPE signal in mammals.

Future experiments can distinguish between these hypotheses. For instance, neural recordings can probe how DAN activity changes over the course of the task, and connectomics can identify other neurons in the system that may be important for the computing of reward expectation. These types of experiments are easily doable in the *D. melanogaster* model. Paired with further modeling efforts and the foraging framework we developed, the fly MB promises to be a system in which we can understand decision-making at a level of detail that is presently unparalleled in systems neuroscience.

## Materials and Methods

The following is a brief description of the paper’s methods. A complete description can be found in *SI Appendix*. Both brief and supplemental methods consist of the same section headings.

### Fly Strains and Rearing.

*D. melanogaster* were raised on standard cornmeal food supplemented with 0.2 mM all-trans-retinal at 25 °C (for Gr64f lines) or 21 °C (for other lines) with 60% relative humidity and kept in dark throughout.

### Cloning.

The Gr64f promoter was amplified using Q5 High-Fidelity 2X- Master Mix (New England Biolabs) from the Gr64f-GAL4 plasmid (84) and cloned into the FseI/EcoRI digested backbone of pBPLexAp65 (27) using NEBuilder HiFi DNA Assembly (New England Biolabs). Four gRNA for the gene Dop1R1 were designed using https://flycrispr.org/target-finder (69). The gRNA were then cloned into pCFD5_5 (85).

### Y-arena.

A detailed schematic of the apparatus is provided in *SI Appendix* and Information 1. A description of the custom MATLAB code (MATLAB 2018b, Mathworks) used to control the Y-arena can be found in *SI Appendix*.

### Circular Olfactory Arena.

Group learning experiments (*SI Appendix*, Fig. S9) were performed in a previously described circular arena (22).

### Behavioral Experiments.

For all experiments in the paper, two or three of the odorants, 3-octanol (OCT) [Sigma-Aldrich 218405], 4-methylcyclohexanol (MCH) [Sigma-Aldrich 153095], and pentyl acetate (PA) [Sigma-Aldrich 109584] were used. Details regarding the instantiation of probabilistic rewards etc. can be found in *SI Appendix*.

### Quantitative Analysis and Behavioral Modeling.

All analysis and modeling were performed using MATLAB 2020b (Mathworks). Details are described in *SI Appendix*.

### Neural Circuit Model of Dynamic Foraging.

We designed two versions of a neural circuit model, inspired by work from Loewenstein and Seung (19), that were used to simulate behavior. The first version aimed to directly replicate the model used by Loewenstein and Seung (*SI Appendix* and Fig. S4*A*). The second version incorporated modifications that made it more appropriate to our task and the mushroom body (Fig. 3 *A*, *Right* and *SI Appendix*).

### Plasticity Requirements of Operant Matching in the Mushroom Body Model.

An expansion of the mathematical proof provided by Loewenstein and Seung’s to incorporate the structure of our task and architecture of the MB can be found in the eponymous section of *SI Appendix*.

### Logistic Regression Model for Estimating Learning Rules.

To determine the learning rules that best predict fly behavior, we designed a logistic regression model that made use of the known relationship between MBON activity and behavior (Fig. 4*A*). The mathematical working of this model can be found in the eponymous section of *SI Appendix*.

## Supplementary Material

Appendix 01 (PDF)

## Data Availability

Matlab code and data have been deposited in Zenodo repositories (86, 87).
