# Supplementary material for: Reward expectations direct learning and drive operant matching in Drosophila
Source: Proc Natl Acad Sci U S A. 2023 Sep 21;120(39):e2221415120. doi: 10.1073/pnas.2221415120 (PMC10523640; doi:10.1073/pnas.2221415120)
Supplement: Supplementary file 1 — Appendix 01 (PDF) [file pnas.2221415120.sapp.pdf]

SI APPENDIX FOR

Reward expectations direct learning and drive operant matching in *Drosophila*

Adithya E. Rajagopalan [a,b], Ran Darshan [a,c], Karen Hibbard [a], James E. Fitzgerald [a], Glenn C. Turner [a,1]

a - Janelia Research Campus, Howard Hughes Medical Institute, Ashburn, VA 20147, USA
b - The Solomon H. Snyder Department of Neuroscience, Johns Hopkins University School of Medicine, Baltimore, MD 21205, USA
c - Department of Physiology and Pharmacology, Sackler Faculty of Medicine; Sagol School of Neuroscience; The School of Physics and Astronomy; Tel Aviv University, Tel Aviv, Israel
1 - Correspondence: turnerg@hhmi.org

Supplemental Materials & Methods

All the data presented in the figures and supplementary figures can be found at the following data repository: https://doi.org/10.5281/zenodo.7449214. The code used to run the Y-arena as well as perform the analysis and production of figures panels can be found in the following code repository: https://doi.org/10.5281/zenodo.7986372

Fly strains and rearing:

Drosophila melanogaster were raised on standard cornmeal food supplemented with 0.2 mM all-trans-retinal at 25℃ (for Gr64f-Gal4 lines - see table) or 21℃ (for other lines - see following table) with 60% relative humidity and kept in dark throughout. The details of all flies used for experiments in this manuscript can be found in the table below:

Genotype	Expression target/reporter description	Bloomington stock number /Reference (if applicable)
w; Gr64f-Gal4/CyO; Gr64f-Gal4/TM3 X 20XUAS-CsChrimson-mVenus attp18	Optogenetic activation of Gr64f expressing sugar sensory neurons	Dahanukar et. al. 2007 (1); Haberkern et. al. 2019 (2) X BDSC:55134 Klapoetke et. al. 2014 (3)
w; +; 58E02-Gal4 X 20XUAS-CsChrimson-mVenus attp18	Optogenetic activation of PAM cluster DAN	BDSC:41347 Jenett et. al. 2012 (4) X BDSC:55134 Klapoetke et. al. 2014 (3)
w; 10XUAS-opCas9wt ZH51C; DopR1-4gRNA JK65C/CyO::TM6B X w; +; 13F02-Gal4 attP2, Gr64LexAp65 JK73A, 13XLexAop-IVS-Syn 21-Chrimson88::tdT/TM3	Optogenetic activation of Gr64f expressing sugar sensory neurons and CRISPR mediated knockout of DopR1 receptors in KCs	New Stocks - see Cloning sub-section below
w; 10XUAS-opCas9wt ZH51C; DopR1-4gRNA JK65C/CyO::TM6B X w; +; Gr64LexAp65 JK73A, 13XLexAop-IVS-Syn 21-Chrimson88::tdT/TM3	Optogenetic activation of Gr64f expressing sugar sensory neurons without expression of cas9 protein needed for knockout of DopR1	New Stocks - see Cloning sub-section below

Cross progeny (2-5 days old) were sorted on a cold plate at around 4℃ and females of the appropriate genotype were transferred to starvation vials. Starvation vials contained nutrient-free 1% agarose to prevent desiccation. Flies were starved between 28 - 42 hrs before being aspirated into the Y-arena for experiments.

Cloning:

To construct Gr64f-LexAp65, we PCR amplified the Gr64f promoter from the Gr64f-GAL4 plasmid (1) using Q5 High-Fidelity 2X Master Mix (New England Biolabs) and cloned into the FseI/EcoRI digested backbone of pBPLexAp65 (5) using NEBuilder HiFi DNA Assembly (New England Biolabs). Primer sequences were:

NEB_GR64f_fwd	GAGGCCCTTTCGTCTTCAAGAATTCCAGCGATTGTCTCTTAGCTGTTAAAATC
NEB_GR64f_rev	CCCCGGGCGAGCTCGGCCGGCCCCTAGGACCTGCTGGGGTAAAC

Four gRNA for the gene Dop1R1 were designed using https://flycrispr.org/target-finder (6). The gRNAs were then cloned into pCFD5_5 using a previously described protocol (7).

Dop1R1 gRNA target sites (5'-3')

Dop1R1 gRNA 1	GACATCCAACTGCTGACAAA
Dop1R1 gRNA 2	GCTGCAGCTCACGACCGCCA
Dop1R1 gRNA 3	CGTGGAATTCGTGGAGAATC
Dop1R1 gRNA 4	ACTGGTGTGATTCCCGCCGA

Primer sequences were:

f.PCR1-4gRNA-Dop1R1	GCGGCCCGGGTTCGATTCCCGGCCGATGCGACATCCAACTGCTGACAAA GTTTTAGAGCTAGAAATAGCAAG
r.PCR1-4gRNA-Dop1R1	TGGCGGTCGTGAGCTGCAGCTGCACCAGCCGGGAATCGAACCC
f.PCR2-4gRNA-Dop1R1	GCTGCAGCTCACGACCGCCAGTTTTAGAGCTAGAAATAGCAAG
r.PCR2-4gRNA-Dop1R1	GATTCTCCACGAATTCCACGTGCACCAGCCGGGAATCGAACCC
f.PCR3-4gRNA-Dop1R1	CGTGGAATTCGTGGAGAATCGTTTTAGAGCTAGAAATAGCAAG
r.PCR3-4gRNA-Dop1R1	ATTTTAACTTGCTATTTCTAGCTCTAAAACTCGGCGGGAATCACACCAGTTGCACCA GCCGGGAATCGAACCC

Transgenic injections were performed by Genetivision using fC31 integrase mediated integration into attP dock sites. Gr64f-LexAp65 was integrated into P{CaryP}JK73A and the Dop1R1 gRNA construct was integrated into P{CaryP}JK65C.

Y-arena:

Single fly behavior experiments were performed in an olfactory Y-arena designed in collaboration with the Janelia Experimental Technology team (jET).

Apparatus design:

A detailed description of the apparatus is provided in Supplementary Information 1. The Y chamber consists of two layers of white translucent plastic. The bottom is a single continuous circular layer and serves as the floor of the Y that flies navigate. The top is a circular layer with a Y shaped hole in the middle that serves as the walls. The length of each arm from center to tip is 5 cm and the width of each arm is 1 cm. These two layers are placed underneath an annulus of black aluminum. A transparent glass disk is located in the center of this annulus and acts as the ceiling of the Y, allowing for video recording of experiments. This transparent disk is rotatable and contains a small hole used to load flies. The black annulus houses three clamps that lock the circular disk in place. All three layers are held together and made airtight with the help of 12 screws that connect the layers.

The Y chamber is mounted above an LED board that provides infrared illumination to monitor the fly's movements, and red light for optogenetic activation. The LED board consists of a square array of red (617 nm peak emission, Red-Orange LUXEON Rebel LED, 122 lm at 700mA, 1.9mW/cm^2) and infrared (IR) LEDs that shine through an acrylic diffuser to illuminate flies. Fly movements were recorded at ~5Hz from above the Y using a single USB3 camera (Flea3, model: FL3-U3-13E4M-C, with longpass filter of 800 nm).

Each arm of the Y has a corresponding odor delivery system, capable of delivering up to 5 odors (modified from 8). For our experiments, olfactometers injected air/odor streams into each arm at a flow rate of 100 ml/min. A crisp boundary between odors and air is formed at the center of the Y (Fig. S1A). Odors and concentrations used for each experiment are detailed in the behavioral experiments section of the Methods. The center of the Y contains an exhaust port connected to a vacuum, which was set at 300 ml/min using a flow meter (Dwyer, Series VF Visi-Float® acrylic flowmeter) - matching total input flow in our experiments.

Fly tracking and operation:

We wrote custom MATLAB code (MATLAB 2018b, Mathworks) to control the Y-arena and run experiments. The data collected by the USB3 camera was loaded into MATLAB in real time and the fly's location was identified using the MATLAB image processing toolbox as follows. A background image was calculated just before beginning the experiment by averaging multiple frames as the fly moved around in the Y. This background was subtracted from the frame being processed and the resulting image was thresholded, leaving the fly as a white shape on a black background. The location of the centroid of the fly was estimated using MATLAB's *bwconncomp* and *regionprops* functions. If the fly was located in one of the reward zones, a trial was deemed complete, and reward was provided by switching on the red LEDs as defined by the reward contingencies of the task. The arena was then reset, air pumped into the chosen arm and odors randomly reassigned to the two other arms (Fig. 1B). The location of the fly, along with other information, such as reward presence and odor-arm assignments, were saved as a .mat file for further analysis. All analysis in Figures 1, 2 and 3 were based on this information.

Circular olfactory arena:

Group learning experiments in Fig. S9 were performed in a previously described circular olfactory arena (8).

Behavioral experiments:

Odorant information:

For all experiments in the paper, two or three of the following odorants were used to form cue-reward relationships:

1. 3-octanol (OCT) [Sigma-Aldrich 218405]. *Y-arena:* diluted in paraffin oil [Sigma-Aldrich 18512] at a 1:500 concentration and then air-diluted to a fourth of this concentration. *Circular olfactory arena:* diluted in paraffin oil at a 1:1000 concentration in the circular arena.

2. 4-methylcyclohexanol (MCH) [Sigma-Aldrich 153095]. *Y-arena:* diluted in paraffin oil [Sigma-Aldrich 18512] at a 1:500 concentration and then air-diluted to a fourth of this concentration. *Circular olfactory arena:* diluted in paraffin oil at a 1:750 concentration in the circular arena.

3. Pentyl acetate (PA) [Sigma-Aldrich 109584]. *Y-arena:* diluted in paraffin oil [Sigma-Aldrich 18512] at a 1:5000 concentration and then air-diluted to a fourth of this concentration.

Y-arena behavioral task structure and design:

The general structure of these experiments starts by inserting a fly randomly into one of the three arms of the Y via aspiration. This arm is injected with a clean airstream, and the odor choices are assigned randomly to the other two arms. This randomization ensures there is no consistent spatial relationship/component to the task. Once a fly reaches an odor arm and travels down to the choice zone, reward is delivered via a 500ms flash of red LED (617 nm, 1.9mW/cm^2) to activate the appropriate reward-related neurons. This constitutes one trial. The arena then resets, the arm chosen by the fly switches to clean air and the odor options are again randomly assigned to the other two arms, and the next trial commences. Trials are strung together into blocks consisting of 60 or 80 trials, depending on the paradigm.

Whenever probabilistic rewards were included in a task, reward baiting was incorporated as follows. On every trial within a block, there is a constant probability that reward will be delivered when a fly makes a particular odor choice. However, once a reward is scheduled to be delivered for a given choice, that state persists until that odor is chosen by the fly; in other words, the odor becomes 'baited with reward' until chosen. Note that this type of reward schedule means that the likelihood an odor cue yields a reward increases over time if it is unchosen for many trials; this design choice is to reflect the replenishment of resources that would occur in a natural foraging environment.

Our terminology designates experiments according to the reward probabilities associated with each odor. For example, 100:0 indicates that one odor was rewarded on 100% of the trials it was chosen, while the other option was never rewarded; this is a non-probabilistic task as in Fig 1. By contrast, 80:20 indicates that one odor is baited with reward with 80% probability, while the other option is baited 20% of the time, and the task is entirely probabilistic.

When we evaluated whether flies learn two different cue-reward pairings, one high probability, one low probability in Fig. 1G and Fig. S1G, we had to use three different odors, OCT, MCH and PA. In these experiments PA was always an unrewarded odor cue, while OCT and MCH were arbitrarily assigned high (80%) and low (40%) reward baiting probability. On each trial, flies were presented with a choice of either OCT versus PA or MCH versus PA. These choices were delivered in alternation for a block of 80 unrewarded total trials to assess naive odor preference. This was followed by an 80-trial block with reward baiting as above. For each fly we arbitrarily assigned which odor (OCT or MCH) was associated with high reward probability, to ensure a balance across the dataset.

The dynamic foraging task in e.g. Fig. 2 was adapted from monkey and mouse versions (9-12), and used a three block structure where reward baiting probabilities were constant within a given 80-trial block, and changed between different blocks. We used reward baiting probabilities of 50:50, 33:67, 20:80 and 11:89; in a subset of experiments, we delivered lower net reward at the same ratios: 25:25, 16.5:33, 10:40 and 5.5:44.5.

Circular olfactory arena behavioral task structure and design:

A schematic of the task performed in the circular arena is shown in Fig. S9A. OCT and MCH were used as odors for these experiments. Odors were presented sequentially (and separated in time) for one minute each, with one of the odors paired with reward. To mimic the relationship between odor time and reward time experienced by the fly in the Y-arena, 1 sec of reward (red light, 617nm, 2.3mW/cm^2), was provided after every 3 seconds of odor experience. Flies were finally tested by dividing the circular arena into four quadrants with two opposite quadrants receiving one odor and the other two quadrants receiving the other.

Quantitative analysis and behavioral modeling:

All analyses and modeling were performed using MATLAB 2020b **(**Mathworks). We used nonparametric statistical tests when quantifying statistical significance in all cases. The Mann-Whitney test was used when testing hypotheses with unpaired samples. The Wilcoxon signed-rank test was used with paired samples. Specific descriptions of the hypotheses being tested are provided in the results and figure legend in each case.

Analysis of fly movement and choices in the Y-arena:

The *(x,y)* coordinates of the fly were analyzed to calculate: i) the distance of the fly from the center of the Y; ii) when the fly entered and exited a given odor arm; and iii) the time taken per trial to

enter into the reward zone at the end of an odorized arm. These quantities were then used to produce the plots in Fig. 1, 2, 5 and Fig. S1, 2.

Distance from center was calculated by projecting the (*x,y*) location of the fly (pt) onto a skeleton of the Y and this metric was used when plotting location over time plots (example shown in Fig. 1C) . Here the subscript t denotes the time point at which the (*x,y*) location was observed. The skeleton consisted of three lines running down the middle of each arm to the center of the Y (v_0). Based on which arm the fly was located in, its (*x,y*) position was projected onto the appropriate (i^{th}) skeleton line using the following equations for projecting a point onto a line,

$$D_i = \frac{\|b \cdot a\|}{\|a\|}, \tag{1}$$

where $b = pt - v_0$, $a = v_i - v_0$ and v_i is the (x,y) coordinates of the end of the ith arm. The entries/exits of a fly into/from a particular odorant or air were estimated by tracking the region that the fly was located in at every time point and comparing it to the known odor-arm identity map (stored in the experiment .mat file). A turn (reversal) was considered to have been made whenever a fly entered an odor and then exited this odor without reaching the reward zone. An approach was considered to have been made whenever a fly entered an odor arm and then traveled all the way into the reward zone of that same arm without ever exiting it.

To calculate the time taken per trial, we made use of the timestamp vector that we saved along with the (*x,y*) vector. Time taken from the entire trial was calculated by subtracting the timestamp for the frame that the previous trial was completed by the timestamp of the frame when the current trial was completed. Time taken from first exit of the air arm was calculated by subtracting the timestamp of the frame that the fly first exited the air arm after a trial began by the timestamp of the frame when the current trial was completed.

Choices themselves were determined by identifying the arm in which the fly crossed into the reward zone and mapping that arm to its assigned odor on that trial. Once choices were determined we could calculate two important metrics. Choice ratio, defined as the ratio between the number of choices made towards option A to the number of choices made towards option B, and reward ratio, defined as the ratio between the number of rewards received upon choosing option A to the number of rewards received upon choosing option B. These ratios were calculated on one of two timescales, i) the ratio over an entire block of 80 trials where baiting probabilities were constant, or ii) the ratio in a ten-trial moving window over the entire 240 trials of the experiment. The undermatching index used in Fig. 4E,F was defined as the mean square error between the instantaneous choice ratio and reward ratio curves produced for each fly.

Analysis of fly location in the circular arena:

Videos of a fly's movements in the Y-arena were read into MATLAB frame by frame and the location of the fly's centroid was identified using the MATLAB image processing toolbox. Once identified, the number of flies in each quadrant was used to calculate the preference index (PI) metric on a per frame basis. PI is defined as the difference between the number of flies in each pair

of odor-matched quadrants divided by the total number of flies. Time-averaged PIs could then be calculated by taking the average of the PIs from each individual frame.

Logistic regression to estimate influence of past rewards and choices on behavior:

To estimate the role of choice and reward histories in determining fly choices in the dynamic foraging task, we fit the following logistic regression to each fly's choice sequence as

$$\ln\left(\frac{P(C(t)=1)}{1-P(C(t)=1)}\right) = \sum_{i=1}^{T} \beta_i^R \cdot R(t-i) + \sum_{i=1}^{T} \beta_i^C \cdot C(t-i) + \beta_0 , \tag{2}$$

where t is the present trial and i is the variable used to iterate over the past T trials. $C(t) = 1$ if the chosen odor was OCT and -1 if the chosen odor was MCH. $R(t) = 1$ if chosen OCT option produced reward, -1 if chosen MCH option produced reward, and 0 otherwise. β_0 represents the weight assigned to the bias term, β_i^C represents the weight assigned to the ith past choice and β_i^R represents the weight assigned to the ith past reward. We chose to look at the past T = 15 trials to align with previous studies (12, 13). The regression coefficients generated were 10-fold cross-validated, and the regression model included an elastic net regularization (MATLAB function - lassoglm). The weight of lasso versus ridge optimization was set to 0.1 as this value provided best fits to behavior. These fly-specific regression coefficients could be combined with the flies reward and choice histories to predict trial choice probability and estimate the log-likelihood (ℓ) and percent deviance explained (PD)

$$\ell = \sum_{i=1}^{T_{max}} \ln\left(\sum_{j=1}^{2} p(x_{ji}) \cdot x_{ji}\right) , \tag{3}$$

$$PD = \frac{-2 \cdot \ell_{null} + 2 \cdot \ell_{model}}{-2 \cdot \ell_{null}} \tag{4}$$

where T_{max} is the total number of trials in the data being fit, i indexes trials, j indexes possible options, $p(x_{ji})$ is the probability with which the model predicts that choice j occurs on trial i, and x_{ji} is the choice that actually took place on trial i.

Leaky integrator model:

We also developed a leaky integrator model to predict behavior in the dynamic foraging task inspired by earlier work (9). This model determines choices on a given trial by comparing values assigned to each option the agent has to choose between based on choice and reward history.

The values (Q) were calculated for a given trial t using the following equations. If OCT is chosen by the model, values are updated according to

$$Q_{OCT}(t+1) = \tau \cdot Q_{OCT}(t) + (1-\tau) \cdot R(t) , \tag{5}$$

$$Q_{MCH}(t+1) = \tau \cdot Q_{MCH}(t)\,, \tag{6}$$

where τ is a constant related to the learning rate. Similarly, if MCH is chosen by the model, values are updated according to

$$Q_{OCT}(t+1) = \tau \cdot Q_{OCT}(t)\,, \tag{7}$$

$$Q_{MCH}(t+1) = \tau \cdot Q_{MCH}(t) + (1-\tau) \cdot R(t)\,. \tag{8}$$

These values are then compared and passed through a sigmoidal nonlinearity to determine the probability of each choice,

$$P(C(t) = OCT) = \frac{1}{1 + e^{-\beta \cdot (Q_{OCT}(t) - Q_{MCH}(t))}} \tag{9}$$

The probability of choosing MCH was one minus that of OCT. The probability generated by this operation is compared with a value drawn from a uniform distribution over the [0,1] interval to determine whether the resulting choice is OCT or MCH. These predicted choices could be compared to fly behavior to compute the model's percentage deviance explained. The parameters β and τ are fit for each fly so as to maximize the percentage deviance explained (values of these parameters can be seen in Fig. 2G and Fig. S3A,B).

Win - stay, Lose - switch model:

A third model to predict behavior incorporated information only about the fly's most recent choice, unlike the logistic regression and leaky-integrator alternatives. In this "win-stay, lose-switch" model the agent chooses randomly on the first trial. If the chosen option produces a reward the agent picks the option again on the next trial (stays). If it doesn't produce a reward, the agent picks the other option on the next trial (switches). This procedure repeats to generate a sequence of choices. The accuracy of this model was calculated by observing correctly predicted switches and stays as well as incorrectly predicted switches and stays, shown in Fig. 2D as a probability matrix. To calculate this matrix the model was made to predict the behavior of flies on every trial of the dynamic foraging task. The average values across 18 flies run in the dynamic foraging task is presented in the matrix in Fig. 2D.

Neural circuit model of dynamic foraging:

We designed a neural circuit model, inspired by work from Loewenstein and Seung (14), that was used to simulate behavior in a dynamic foraging task. Two versions of this model were used.

Replicating Loewenstein and Seung's Model:

The first version aimed to directly replicate the model used by Loewenstein and Seung (Fig. S4A). It generated behavior on a trial-by-trial basis in the dynamic foraging task. The number of trials to simulate were input by the user prior to simulation (60, 240 or 2000 trials). The model consisted of two sensory neurons (S_1 and S_2) whose activity was drawn at the beginning of each trial from a normal distribution with mean 1 and standard deviation 0.1. These neurons synapse with weights

(W_1 and W_2) onto two motor neurons (M_1 and M_2). The activity of M_1 and M_2 were compared and the choice was driven by whichever neuron had the larger activity.

Once a choice was made, rewards were provided as determined by the reward contingencies of the task. The weights between S and M were updated after each choice and followed the following rules

$$\Delta W_i(t) = \eta \cdot \tilde{R}(t) \cdot \tilde{S}(t)\,, \tag{10}$$

$$W_i(t) = W_i(t-1) + \Delta W_i(t)\,, \tag{11}$$

where $\tilde{R} = R$ or $\tilde{R} = R - E(R)$ and $\tilde{S}_i = S_i$ or $\tilde{S}_i = S_i - E(S_i)$ based on the learning rule, and i iterates over odors. Note that $E(R)$ and $E(S_i)$ depended on time and were calculated in one of two ways: i) by calculating the mean over the last 10 trials, ii) by filtering the entire history with an exponential filter with exponential timescale of 3.5 trials. The various covariance and noncovariance rules were achieved by selecting the appropriate combinations of $\tilde{R}$ and $\tilde{S}_i$.

Task and mushroom body inspired version:

The second version incorporated modifications to the model that made it more appropriate for the task we designed for fruit flies (Fig.3B). This model consisted of two sensory inputs that represented activity of populations of Kenyon cells (KCs). However, this version of the model looped through odor experiences, rather than looping through trials determined by two-alternative forced choices. Therefore, the activity of the sensory neurons was drawn differently. Rather than both values being drawn from normal distribution with mean 1 and standard deviation $\sigma = 0.1$, this was only true for the odor that was deemed to have been "experienced" by the model on a given odor experience. The activity of the other neuron was drawn from a normal distribution with mean $\alpha = 0.1$ (Fig. 3,4; Fig. S5,6) and standard deviation $\sigma = 0.1$. Here, α represents the similarity, or overlap, between the two inputs. This was included because the KC representations of the two different odors used in our task are thought to have some amount of overlap (15). However, we found that modulating this term did not affect the resulting matching behavior (Fig. S6D-G) and so for Fig. 5, we chose $\alpha = 0$. We also explored incorporating noise covariance between the two sensory inputs (with correlation coefficient c = 0.1), but this correlation was empirically unimportant, and we usually set c = 0.

Another difference is that an odor experience could lead to either an approach (choice) or a turn away. The behavior chosen by the model on any given odor experience depended on the response of the single output neuron incorporated into this model. The activity of this output neuron (M) was the weighted sum of the two inputs. This was then passed through a sigmoidal nonlinearity

$$P(A = Reject) = \frac{1}{1 + e^{\beta \cdot (M-b)}}\,, \tag{12}$$

where β = 4, b = 1 (this value was chosen to encourage exploration at the beginning of learning) and A is the action produced by the model. When A = 0 the odor is always accepted and when A = 1 the odor is always rejected. A random number from the interval [0,1] was drawn and compared

to A to determine whether an approach/choice or turn was made. If a turn was made, no reward was provided, and weights remained unchanged. The model then experienced a new odor and the process repeated. If a choice was made, then a reward was provided based on choice contingencies and weights were updated according to the rules in eqs. 10 and 11. We add the additional constraints that η is negative (synaptic depression) and that weights have a lower bound of 0.

Plasticity requirements of operant matching in the mushroom body model:

Relating operant matching to the covariance of neural activity and reward

We begin by reproducing the key theoretical argument provided by Loewenstein and Seung. Consider a sequence of trials where an animal chooses between two options and receives feedback via reward. Specifying an element in this trial sequence requires three random variables, the choice (C), the reward (R), and the underlying neural activity (N). Note that N is very general in this argument; it can be any quantification of neural activity whose mean depends on choice. We further assume that both options are sometimes chosen, and that neural activity and reward are conditionally independent on choice. Under these assumptions, Loewenstein and Seung show that Herrnstein's operant matching law is satisfied if and only if the covariance between N and R vanishes over the trial sequence.

The proof begins by recalling the definition of covariance,

$$Cov(N, R) = E(\delta N R)\,, \tag{13}$$

where E denotes the expectation over the trial sequence (i.e., C, R and N) and $\delta N = N - E(N)$. By the product rule of probability, $P(C, R, N) = P(C)P(R, N|C)$, so we can rewrite this expectation as

$$Cov(N, R) = E_C\left(E_{R,N|C}(\delta N R)\right) = E_C\left(E_{N|C}(\delta N)E_{R|C}(R)\right)\,, \tag{14}$$

where the subscripts on E denote the probability distributions over which the expectations are computed, and we used the conditional independence assumption, $P(R, N|C) = P(R|C)P(N|C)$, in the second step. Writing out the expectation over choice explicitly, the expression for the covariance becomes

$$Cov(N, R) = P(C = 1)E_{N|C=1}(\delta N)E_{R|C=1}(R) + P(C = 2)E_{N|C=2}(\delta N)E_{R|C=2}(R)\,. \tag{15}$$

To simplify this expression, note that

$$E(\delta N) = E_C\left(E_{N|C}(\delta N)\right) = 0 \implies P(C = 2)E_{N|C=2}(\delta N) = -P(C = 1)E_{N|C=1}(\delta N)\,, \tag{16}$$

where we again used the product rule of probability, and the $\Longrightarrow$ notation means that the lefthand equation implies the righthand one. It follows that

$$Cov(N, R) = P(C = 1)E_{N|C=1}(\delta N)\left(E_{R|C=1}(R) - E_{R|C=2}(R)\right). \quad (17)$$

From this expression, we can conclude that

$$E_{R|C=1}(R) = E_{R|C=2}(R) \Rightarrow Cov(N, R) = 0. \quad (18)$$

The lefthand equation is the matching law, as it says that the expected reward is independent of choice, so the matching law implies a vanishing covariance between neural activity and reward. Moreover, it follows that $E_{N|C=1}(\delta N) \neq 0 \neq E_{N|C=2}(\delta N)$ from the assumption that the average neural activity has a choice dependence (i.e., $E_{N|C=1}(\delta N) \neq E_{N|C=2}(\delta N)$). Consequently, neither $P(C = 1)$ nor $E_{N|C=1}(\delta N)$ is equal to zero, and

$$Cov(N, R) = 0 \Rightarrow E_{R|C=1}(R) = E_{R|C=2}(R) \quad (19)$$

This equation says that the matching law follows from the vanishing covariance of choice-related neural activity and reward. This completes the proof.

Casting the mushroom body model in the framework of Loewenstein and Seung

Our mushroom body model consists of a sequence of odor presentations and accept/reject decisions. Specifying an element in this decision sequence requires four random variables, the odor option experienced (O), the odor-induced neural activity in the KCs (S), the accept-reject decision provided by the MBON (A), and the reward received from this action (R). Reward delivery and plasticity only occur when an odor option is accepted, and we refer to the accepted odor option as the choice (C). Note that C is undefined when the odor is rejected. Therefore, specifying an element in the choice sequence only requires three random variables, S, C, and R. Setting $N = S$, this choice sequence satisfies the assumptions of Loewenstein and Seung's theory. We therefore expect flies to obey Herrnstein's operant matching law if and only if the covariance between KC activity and reward is equal to zero over the choice sequence.

It is important to recognize that the matching law is generally inconsistent with vanishing covariance between KC activity and reward over the decision sequence (rather than the choice sequence). Our assumption that plasticity only occurs following the decision to accept (*i.e.*, the choice) is thus critical for obtaining matching behavior from covariance-based plasticity rules.

Vanishing covariance does not imply matching between more than two alternatives

The preceding analyses assumed binary choices between two options. However, Herrnstein's operant matching law can also be satisfied with more than two options, and the general form of the matching law is

$$E_{R|C=1}(R) = E_{R|C=2}(R) = \cdots = E_{R|C=n}(R), \quad (20)$$

where $n \geq 2$ is the number of options. We can write this condition more succinctly as

$$E_{R|C}(R) = E(R)\,. \qquad (21)$$

Here we show that this more general form of the matching law implies that the covariance between neural activity and reward vanishes. However, the converse is not true, as it's possible for the covariance to vanish without behavior that produces the matching law. The biologically important consequence of this result is that covariance-based plasticity rules may not lead to matching when the animal is deciding between more than two options.

In this more general decision-making task, we express the covariance between the neural activity and reward over the choice sequence as

$$Cov(N,R) = E_C\left(E_{N|C}(\delta N)E_{R|C}(R)\right) = \sum_{c=1}^{n} P(C=c)E_{N|C=c}(\delta N)E_{R|C=c}(R)\,, \qquad (22)$$

where we've made the same conditional independence assumption as in the binary analysis. If the matching law is satisfied, then we can take $E_{R|C=c}(R)$ out of the sum and we find

$$E_{R|C}(R) = E(R) \Rightarrow Cov(N,R) = E(R)\sum_{c=1}^{n} P(C=c)E_{N|C=c}(\delta N) = E(R)E(\delta N) = 0\,. \qquad (23)$$

Therefore, the matching law implies that the covariance between neural activity and reward is zero. To see that the converse need not be true, we construct a specific counter example. Consider the $n = 3$ case where $P(C = c) = 1/3$, $E_{N|C=1}(\delta N) = 2$, $E_{N|C=2}(\delta N) = E_{N|C=3}(\delta N) = -1$, $E_{R|C=1}(R) = 1/2$, $E_{R|C=2}(R) = 1$, and $E_{R|C=3}(R) = 0$. These numbers were constructed to ensure that the covariance between neural activity and reward vanishes,

$$Cov(N,R) = \frac{1}{3}\left(\left(2\cdot\frac{1}{2}\right) - (1\cdot 1) - (1\cdot 0)\right) = 0\,. \qquad (24)$$

Nevertheless, the matching law is not satisfied because the expected reward depends on the choice.

Logistic regression model for estimating learning rules:

To determine the learning rules that best predict fly behavior, we designed a logistic regression model that made use of the known relationship between MBON activity and behavior. This model predicted behavior between input and weights that give rise to MBON activity following the relationships

$$\hat{A}(t) = \sum_{i=1}^{2} W_i(t)\cdot S_i(t)\,, \qquad (25)$$

$$W_i(t) = W_i(0) + \sum_{T|A=0} \Delta W_i(T)\,, \qquad (26)$$

where $\hat{A}(t)$ is the predicted action on odor experience t, $T|A = 0$ indicates all past odor experiences where the fly chose to accept the odor, and $W_i(t)$ represents the synaptic weights associated with neurons representing odor i at time t. Now the change in synaptic weights $\Delta W_i(T)$ depends on the learning rule that is used by the circuit. It was here that we wanted to have the regression model identify the rule that provided the best fit to actual data. To do this we allowed the model to use a learning rule with 4 different terms whose coefficients could be modified,

$$\Delta W_i(T) = -a - b \cdot \tilde{S}_i(T) - c \cdot \tilde{R}(T) - d \cdot \tilde{R}(T) \cdot \tilde{S}_i(T)\,. \tag{27}$$

Here, a, b, c, and d are the coefficients assigned to each component of the learning rule. The regression model takes the sensory stimuli and synaptic weights at a given time as inputs to predict the output action. However, when fitting this model to behavior we have only sensory stimulus and reward information readily available. We therefore used eq. 26 and 27 to convert synaptic weights and sensory stimuli to inputs that consisted of sensory stimuli and rewards and a constant input that serves as a bias term. The resulting inputs could be represented as

$$I_0(T) = 1\,, \tag{28}$$

$$I_1(T) = \sum_{i=1}^{2} (T-1) \cdot S_i(T)\,, \tag{29}$$

$$I_2(T) = \sum_{i=1}^{2} S_i(T) \cdot \sum_{t=1}^{T-1} \tilde{S}_i(t)\,, \tag{30}$$

$$I_3(T) = \sum_{i=1}^{2} S_i(T) \cdot \sum_{t=1}^{T-1} \tilde{R}_i(t)\,, \tag{31}$$

$$I_4(T) = \sum_{i=1}^{2} S_i(T) \cdot \sum_{t=1}^{T-1} \tilde{S}_i(t) \cdot \tilde{R}_i(t)\,. \tag{32}$$

The coefficients assigned to each of the five inputs $(W_i(0), a, b, c, d)$ could then be used to identify the learning rule that the model predicted as the best estimate for producing the behavior that was tested. Of course, the values of these coefficients varied from fly to fly. To examine if pairs of coefficients changed in a correlated manner across flies, we estimated the correlations between the terms by using the Matlab function corrcoef, that produces a matrix of correlation coefficients.

Figure S1

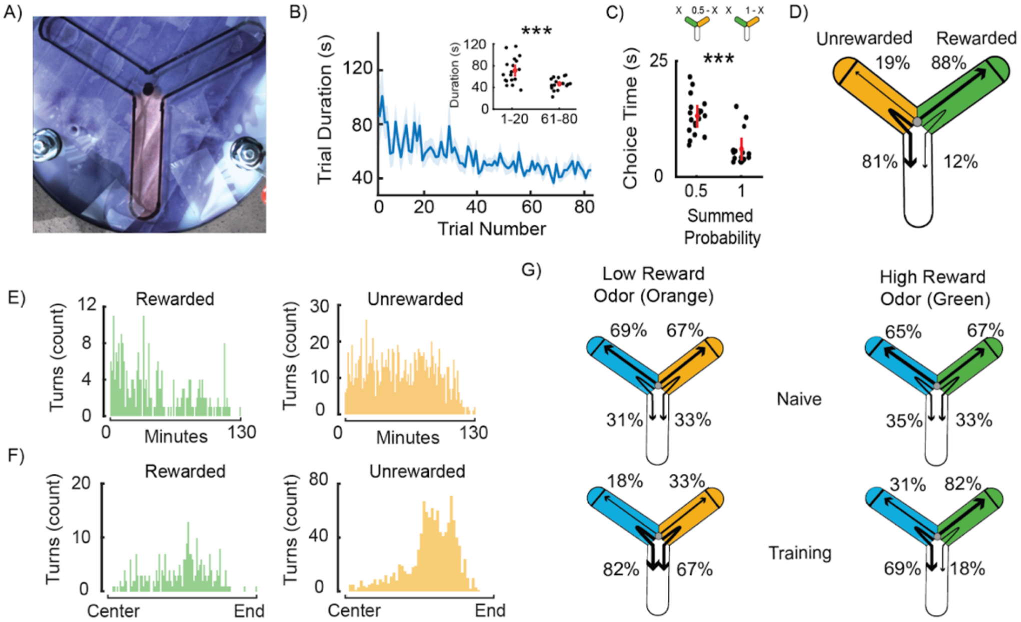

Figure S1: Further quantification of learning of multiple probabilistic cue-reward associations in the novel Y-arena

A A measurement of odor boundaries in the Y-arena. Moist blue litmus paper was placed in the Y-arena while one arm was filled with carbon dioxide. This caused the color of the litmus paper to change, providing an estimate of how odor boundaries are formed at the center of the Y.

B The time taken to make a choice decreases once reward is made available to the fly (mean +/- SEM). *Inset:* Average trial time for the first 20 trials is longer than the average trial for the last 20 trials (Wilcoxon signed-rank test: $p = 6.292 \times 10^{-4}$, n=18 flies).

C The average choice time across 80 trials, measured from first exit of the air arm till entry into the reward zone, for two different summed probabilities of receiving reward (0.5 or 1). Average choice time decreases as the summed probability increases (Mann-Whitney rank-sum test: $p = 4.7653 \times 10^{-4}$, n = 18 for summed probability = 1; n = 20 for summed probability = 0.5).

D Percentage of odor entries that lead to accept or reject in a 100:0 protocol. Flies show increased rejection of the unrewarded odor (Mann-Whitney rank-sum test: $p = 3.2278 \times 10^{-7}$, n = 18), & decreased rejection of the rewarded one (Mann-Whitney rank-sum test: $p = 3.2166 \times 10^{-7}$, n = 18).

E Histogram showing the number of rejects over time for rewarded and unrewarded odor choices. Rejects decrease over time for the rewarded odor (Wilcoxon signed rank test: p =0.0171, n = 18, comparing 15-65 with 66-115 minutes - to exclude large time values when many flies had already finished the task), but not the unrewarded one (Wilcoxon signed rank test: p = 0.1839, n = 18).

F Histogram showing the number of reversals as a function of distance along the odorized arm. The demarcation of “End” on the x-axis represents entry into the reward zone.

G The average percentages of accepting and rejecting each odor - high-rewarding (green), low-rewarding (orange) and unrewarded (blue) are graphically represented in a schematic of the Y-arena (n=10 flies). Flies increasingly accepted the high-rewarding odor (Mann-Whitney rank-sum test: $p = 1.8267 \times 10^{-4}$, n = 10), and displayed an increased probability of rejecting both low-rewarding and unrewarded odors, as compared to naive trials (Fig. S1G left; Mann-Whitney rank-sum test: unrewarded odor: $p = 1.8165 \times 10^{-4}$, n = 10; rewarded odor: $p = 7.6854 \times 10^{-4}$, n = 10).

Figure S2:

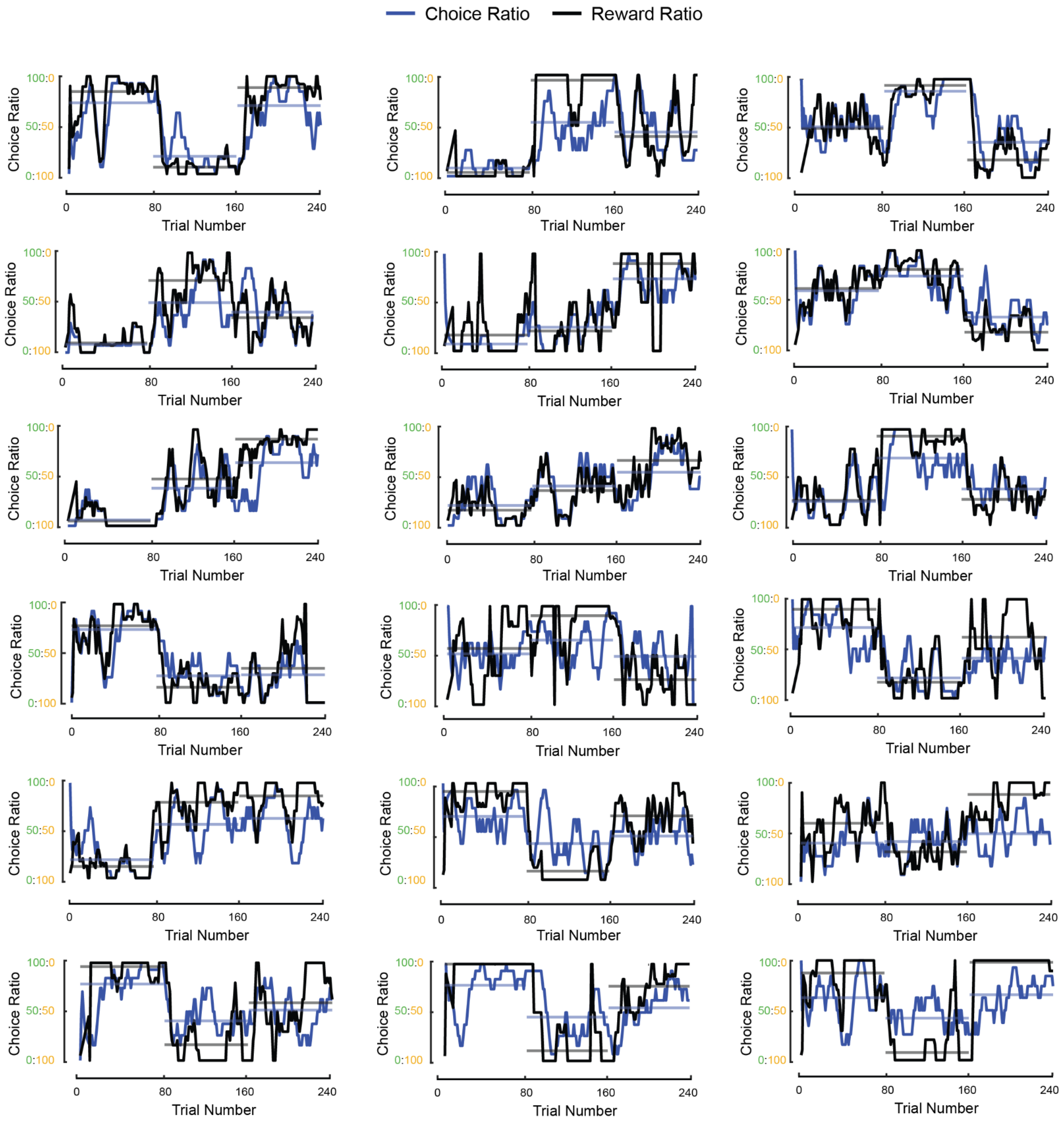

Figure S2: All instantaneous choice ratio and reward ratio plots
Matching of instantaneous choice ratio (blue) and reward ratio (black) in all flies. Curves show 10-trial averaged choice ratio and reward ratio, and horizontal lines the corresponding averages over the 80-trial blocks.

Figure S3

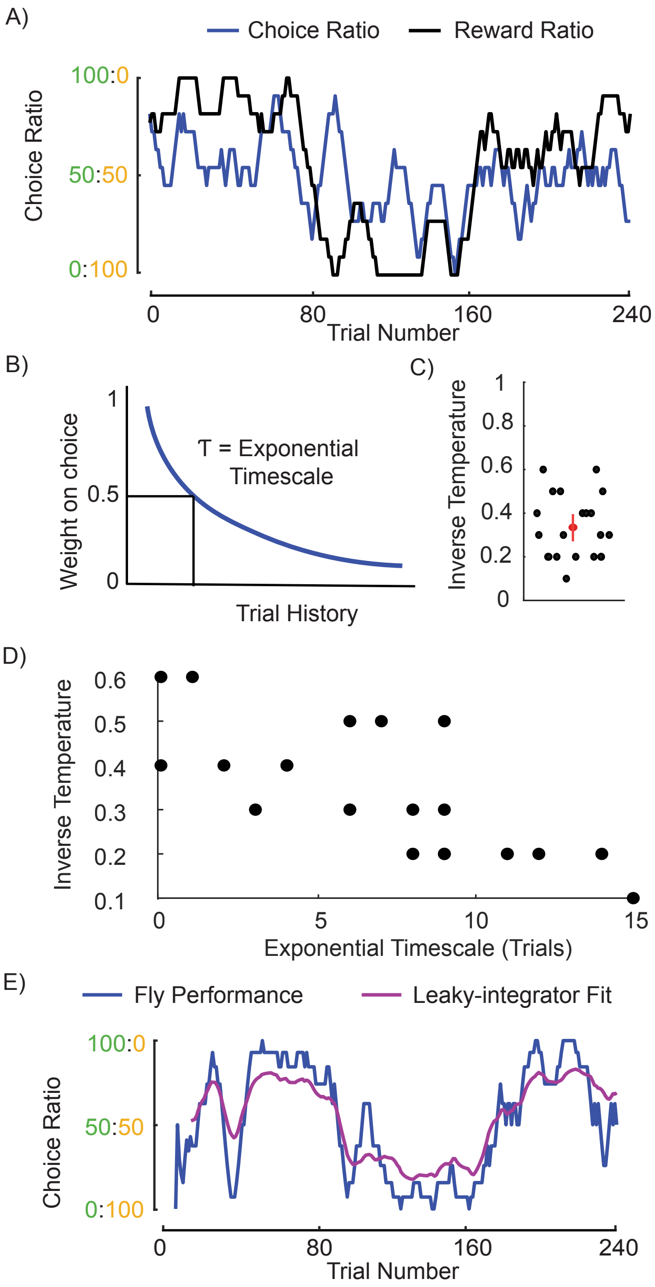

Figure S3: Analysis of the "win-stay lose-switch" and "leaky-integrator" models

A Example choice data generated by the "win-stay; lose-switch" model showing instantaneous choice ratio (blue) and reward ratio (black)

B Schematic representing how the past trial history is weighted to calculate value in the "leaky-integrator" model.

C Estimated inverse temperatures for each fly shown in Fig. S2

D Relationship between exponential timescale and inverse temperature.

E Leaky-integrator model fit (purple) on behavior (blue) from the example fly in Fig. 2A, plotted from the 15th trial onwards to avoid edge effects.

Figure S4

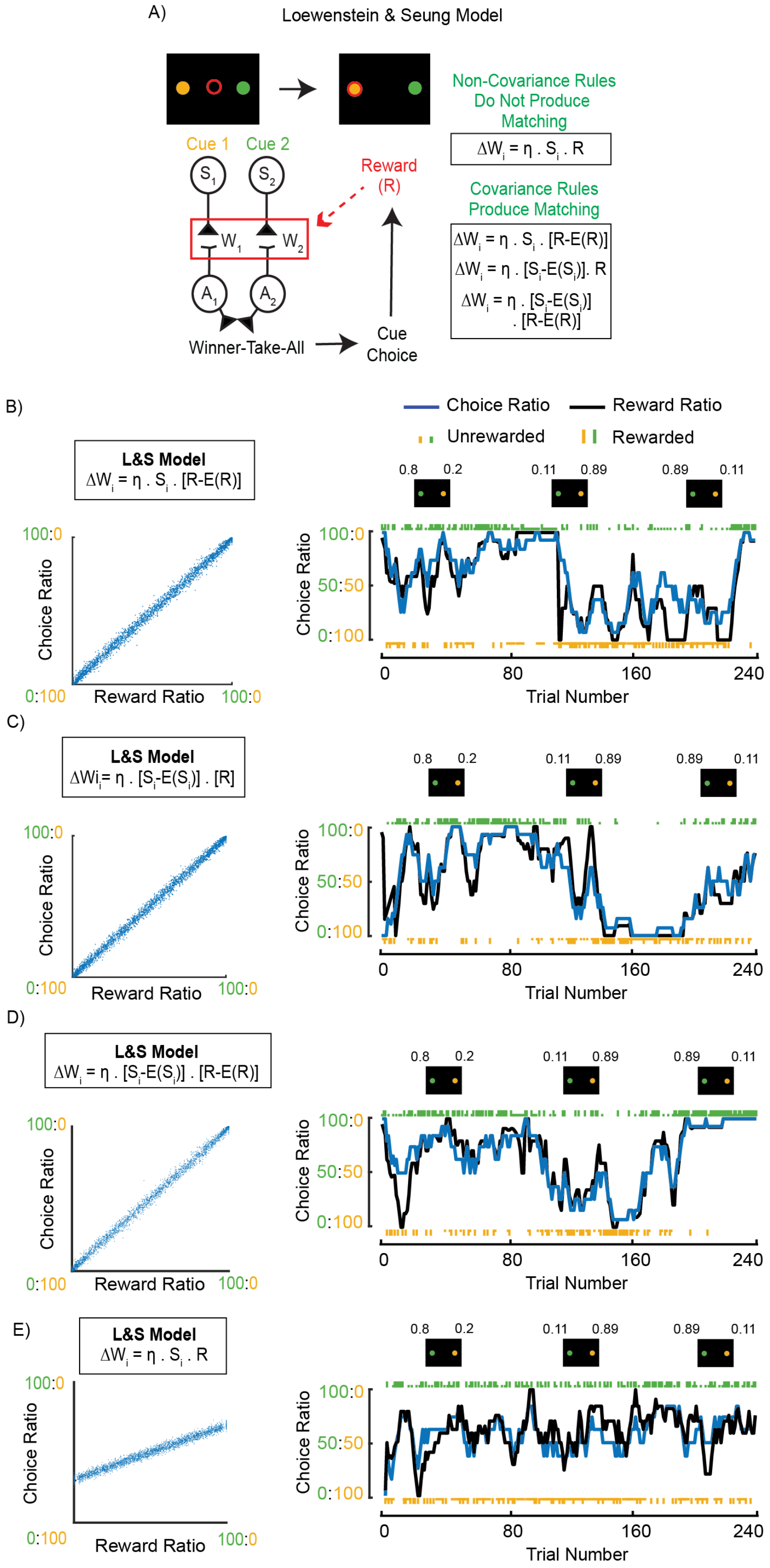
A)
Loewenstein & Seung Model
Non-Covariance Rules Do Not Produce Matching
ΔW_i = η . S_i . R
Covariance Rules Produce Matching
ΔW_i = η . S_i . [R-E(R)]
ΔW_i = η . [S_i-E(S_i)]. R
ΔW_i = η . [S_i-E(S_i)] . [R-E(R)]
Cue 1
Cue 2
S_1
S_2
Reward (R)
W_1
W_2
A_1
A_2
Winner-Take-All
Cue Choice
B)
Choice Ratio
Reward Ratio
Unrewarded
Rewarded
L&S Model
ΔW_i = η . S_i . [R-E(R)]
0.8
0.2
0.11
0.89
100:0
50:50
0:100
Trial Number
0
80
160
240
C)
L&S Model
ΔWi= η . [S_i-E(S_i)] . [R]
D)
L&S Model
ΔW_i = η . [S_i-E(S_i)] . [R-E(R)]
E)
L&S Model
ΔW_i = η . S_i . R

Figure S4: Covariance-based learning rules are necessary for operant matching

A Description of the model developed by Loewenstein and Seung to study the requirements of matching behavior. The neural network consists of sensory neurons S_1 and S_2 that respond to one of the two simultaneously provided stimuli and synapse onto motor neurons A_1 and A_2 via synapses with weights W_1 and W_2. Choices are determined via a winner-take-all computation downstream of motor neurons. Upon choice, weights are updated according to one of the shown plasticity rules (*boxes-right*). Here, S_i is the activity of the ith sensory neuron; R represents the presence or absence of reward; $E(S_i)$ is the mean or expectation of the activity of S_i and E(R) is the expectation of reward.

B *Left:* Block-averaged choice ratio produced by the $S_i \cdot [R-E(R)]$ covariances-based rule (*box*) plotted against reward ratio. The model exhibits matching behavior (slope is 1). *Right:* An example simulation showing the performance in a 3 block task of a model incorporating a covariance-based rule $S_i \cdot [R-E(R)]$. Task reward contingencies are the same as shown for example fly in Fig. 2A.

C Same as (B), but simulated with a $[S_i-E(S_i)] \cdot R$ rule. *Left:* The model exhibits matching behavior (slope is 1). *Right:* performance in a 3 block task where reward contingencies are the same as shown for the example fly in Fig. 2A.

D Same as (B), but simulated with a $[S_i-E(S_i)] \cdot [R-E(R)]$ rule. *Left:* The model exhibits matching behavior (slope is 1). *Right:* performance in a 3 block task where reward contingencies are the same as shown for the example fly in Fig. 2A.

E Same as (B), but simulated with a noncovariance ($S_i \cdot R$) learning rule using the task and circuit structure of the Loewenstein and Seung model shown in Fig. S4. *Left:* The model produces behavior that does not show matching (slope < 1). *Right:* performance in a 3 block task does not accurately replicate fly behavior.

Figure S5

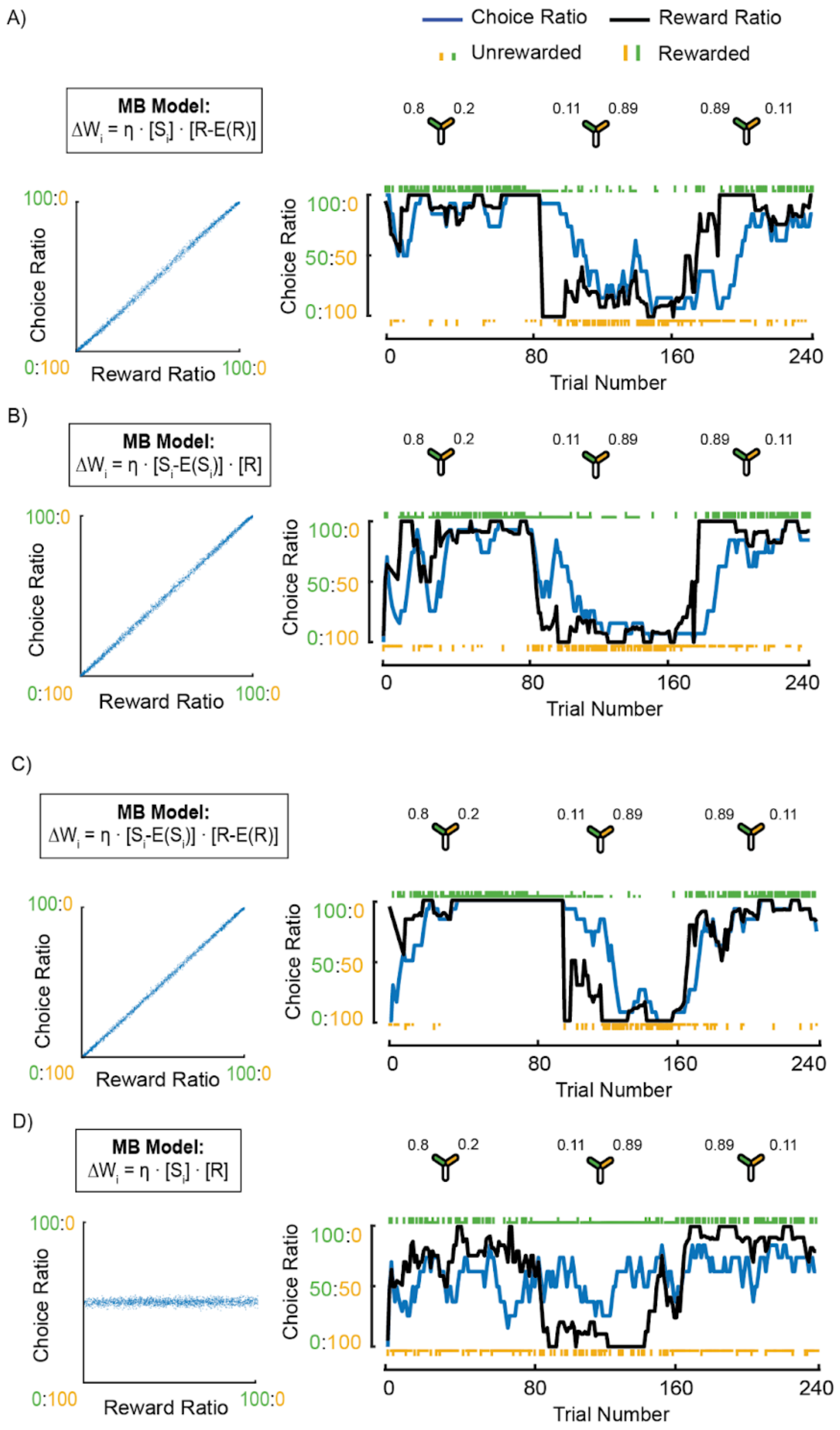

A)
Choice Ratio
Reward Ratio
Unrewarded
Rewarded
MB Model:
ΔW_i = η · [S_i] · [R-E(R)]
0.8 0.2
0.11 0.89
0.89 0.11
100:0
Choice Ratio
0:100
Reward Ratio
100:0
100:0
50:50
0:100
0
80
160
240
Trial Number
B)
MB Model:
ΔW_i = η · [S_i-E(S_i)] · [R]
C)
MB Model:
ΔW_i = η · [S_i-E(S_i)] · [R-E(R)]
D)
MB Model:
ΔW_i = η · [S_i] · [R]

Figure S5: Models using covariance-based learning rules produce behavior more similar to real fly behavior

A *Left:* Block-averaged choice ratio produced by the $S_i \cdot [R-E(R)]$ covariances-based rule (*box*) plotted against reward ratio. The model exhibits matching behavior (slope is 1). *Right:* An example simulation showing the performance in a 3 block task of a model incorporating a covariance-based rule $S_i \cdot [R-E(R)]$. Task reward contingencies are the same as shown for the example fly in Fig. 2A.

B Same as (A), but simulated with a $[S_i-E(S_i)] \cdot R$ rule. *Left:* The model exhibits matching behavior (slope is 1). *Right:* performance in a 3 block task where reward contingencies are the same as shown for the example fly in Fig. 2A.

C Same as (A), but simulated with a $[S_i-E(S_i)] \cdot [R-E(R)]$ rule. *Left:* The model exhibits matching behavior (slope is 1). *Right:* performance in a 3 block task where reward contingencies are the same as shown for the example fly in Fig. 2A.

D Same as (A), but simulated with a noncovariance ($S_i \cdot R$) learning rule. *Left:* The model produces behavior that does not show matching (slope < 1). *Right:* performance in a 3 block task does not show matching of choice and reward ratio.

Figure S6

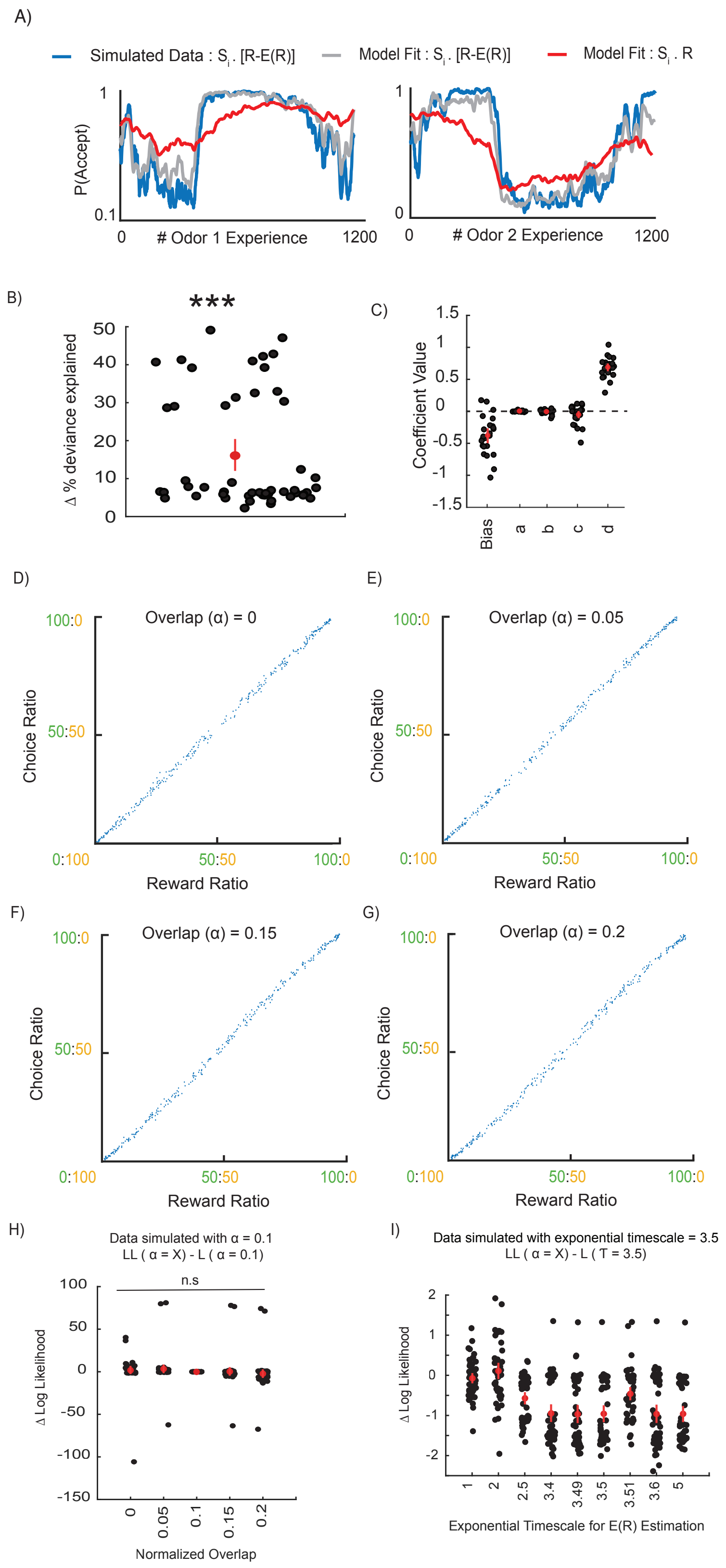
A)
Simulated Data : S_i . [R-E(R)]
Model Fit : S_i . [R-E(R)]
Model Fit : S_i . R
P(Accept)
# Odor 1 Experience
# Odor 2 Experience
B)
***
Δ % deviance explained
C)
Coefficient Value
Bias
a
b
c
d
D)
Overlap (α) = 0
E)
Overlap (α) = 0.05
F)
Overlap (α) = 0.15
G)
Overlap (α) = 0.2
Choice Ratio
Reward Ratio
100:0
50:50
0:100
H)
Data simulated with α = 0.1
LL (α = X) - L (α = 0.1)
n.s
Δ Log Likelihood
Normalized Overlap
I)
Data simulated with exponential timescale = 3.5
LL (α = X) - L (T = 3.5)
Δ Log Likelihood
Exponential Timescale for E(R) Estimation

Figure S6: Extent of sensory overlap does not affect the behavior of our model

A Example simulated data in an experiment that consisted of 3 blocks of 80 trials each with baiting probabilities changing between blocks, showing the probability of accepting odor 1 *(left)* and odor 2 *(right)* (blue), simulated using a covariance-based rule with reward expectation, and fit using an MB-inspired regression model (A) that incorporates either the same rule (gray), or a noncovariance rule (red). The predictions resulting from the model using the covariance-based rule is a better fit for the simulated data. Note: While the number of trials is 240, what we are plotting is the probability of accepting an odor upon experiencing it. Flies reject odors a lot and so the number of odor experiences is much more than 1 per trial leading to the x-axis of these plots having more than 1000 experiences.

B Change in the percentage deviance explained between noncovariance and a covariance-based model. Both models are used to predict behavior simulated using a covariance-based rule (as in A). The change in goodness of fit is computed by subtracting the percentage deviance explained of the noncovariance-based regression models predictions from the predictions of the model with covariance-based rule. This is plotted for each simulation (n = 50). The covariance-based rule better fits the simulated behavior than the noncovariance-based rule (Wilcoxon signed-rank test: $p = 6.7595 \times 10^{-9}$)

C Regression coefficients assigned to each term of the learning rule when the MB-inspired regression model using a covariance-based rule with reward expectation was fit to the simulated behavior. Note the non-zero weight of the c and d terms.

D Block-averaged choice ratios versus reward ratios (n = 300 simulations) from data simulated using a covariance-based rule with reward expectation are plotted against each other. Sensory overlap = 0.

E Block-averaged choice ratios versus reward ratios (n = 300 simulations) from data simulated using a covariance-based rule with reward expectation are plotted against each other. Sensory overlap = 0.05.

F Block-averaged choice ratios versus reward ratios (n = 300 simulations) from data simulated using a covariance-based rule with reward expectation are plotted against each other. Sensory overlap = 0.15.

G Block-averaged choice ratios versus reward ratios (n = 300 simulations) from data simulated using a covariance-based rule with reward expectation are plotted against each other. Sensory overlap = 0.2.

H Change in percentage deviance explained, computed by subtracting the percentage deviance explained of the covariance-based regression model with overlap = 0.1, from that of regression models with covariance-based rules and different amounts of overlap (0,0.05,0.15,0.2). Points are plotted for each simulation (n = 50). Simulations were run with overlap = 0.1.

I Change in percentage deviance explained, computed by subtracting the percentage deviance explained of the covariance-based regression model with exponential timescale of 3.5, from that of regression models with covariance-based rules and different exponential timescales. Points are plotted for each simulation (n = 50). Simulations were run with exponential timescale of 3.5.

Figure S7

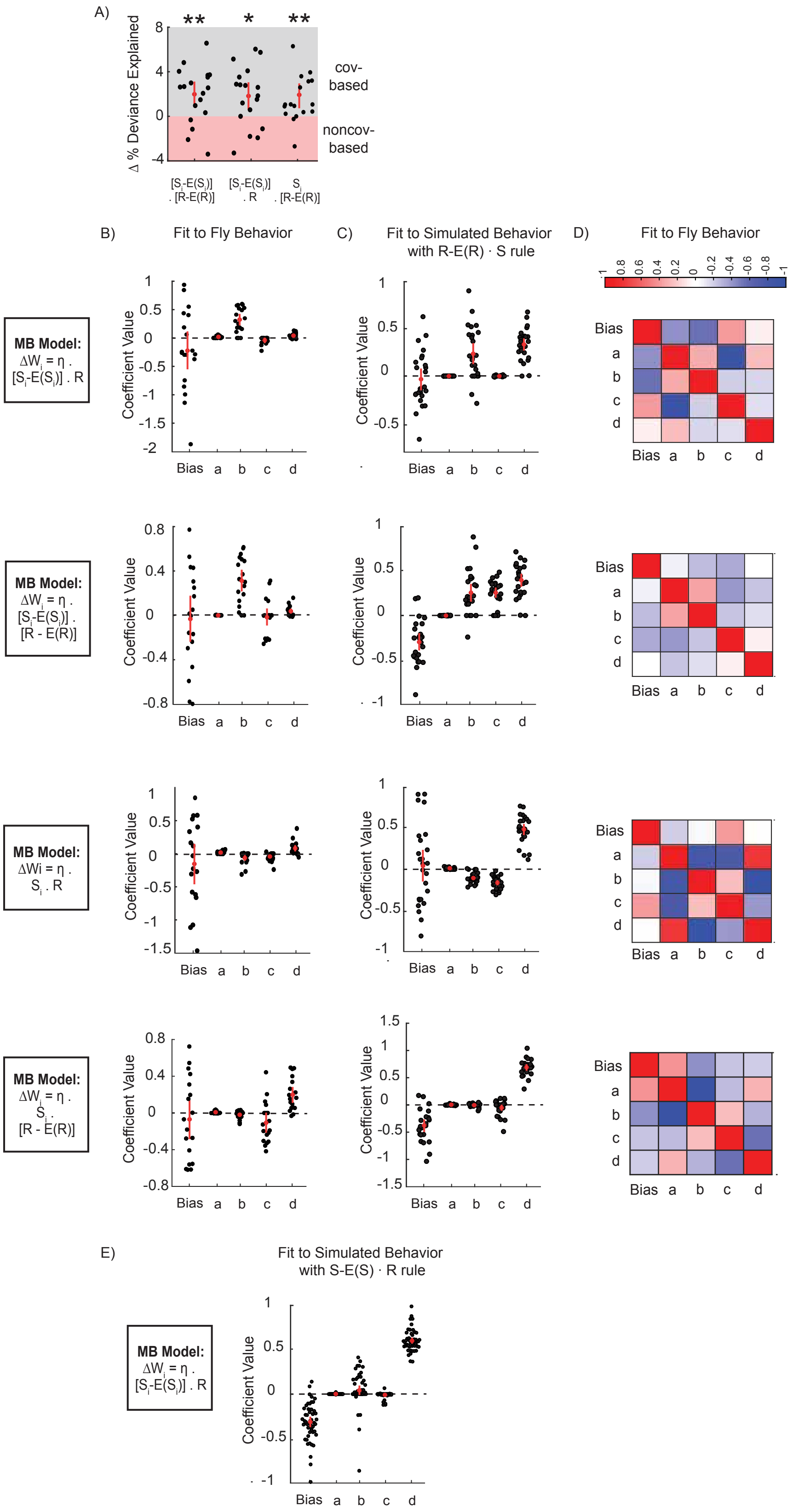
A)
Δ % Deviance Explained
cov-based
noncov-based
B) Fit to Fly Behavior
C) Fit to Simulated Behavior with R-E(R) · S rule
D) Fit to Fly Behavior
MB Model: ΔWi = η . [Si-E(Si)] . R
MB Model: ΔWi = η . [Si-E(Si)] . [R - E(R)]
MB Model: ΔWi = η . Si . R
MB Model: ΔWi = η . Si . [R - E(R)]
Coefficient Value
Bias a b c d
E) Fit to Simulated Behavior with S-E(S) · R rule
MB Model: ΔWi = η . [Si-E(Si)] . R

Figure S7: Covariance-based learning rules are better predictors of individual choice behavior

A Change in percentage deviance explained, computed by subtracting the percentage deviance explained of the noncovariance-based model from three models with covariance-based rules when fit to fly behavior (*left:* incorporating both stimulus and reward expectations; *center:* incorporating just stimulus expectation, *right:* incorporating just reward expectation - same as Fig. 4C) plotted for each fly ($n = 18$). Covariance-based rules were more predictive of fly behavior on average (Wilcoxon signed-rank test: *left*, $p=0.0074$; *center*, $p = 0.0168$, *right, p* = 0.0018).

B Regression coefficients assigned to each term of the learning rule when the MB-inspired regression model was fit to the flies' behavior. All four flavors of model were fit and are indicated on the left.

C Regression coefficients assigned to each term of the learning rule when the MB-inspired regression model was fit to data simulated with a reward-expectation-based covariance rule. All four flavors of model were fit and are indicated to the left of B.

D Correlation between regression coefficients resulting from MB-inspired regression models fit to flies' behavior. All four flavors of model were fit and are indicated to the left of B.

E Regression coefficients assigned to each term of a covariance-based learning rule with only stimulus expectation, when the MB-inspired regression model was fit to data simulated with the same rule.

Figure S8

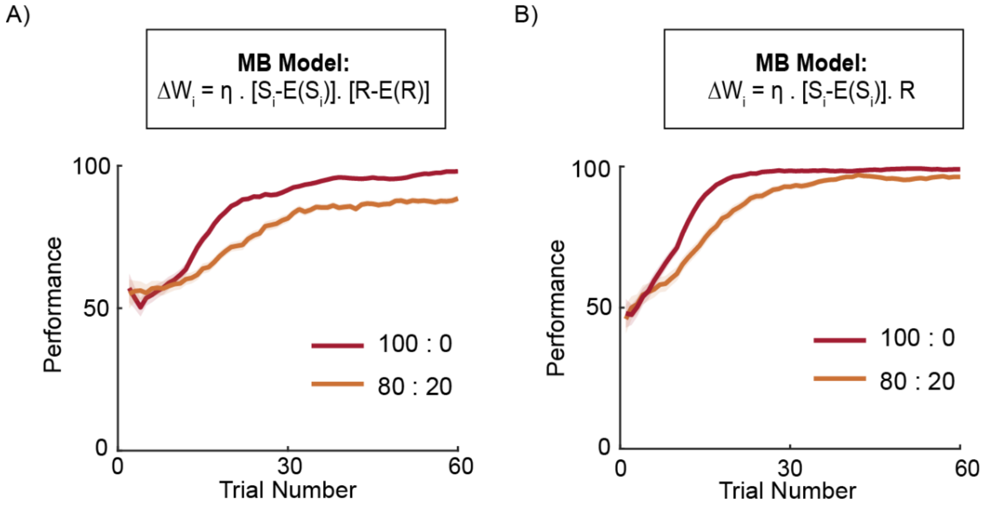

Figure S8: Covariance-based learning rules produce similar behavior in 100:0 and 80:20 tasks

A Simulated instantaneous performance plotted as a function of trial number (defined as the percentage of choices towards the option with higher pre-defined baiting probability in a 10 trial window) of an agent using a covariance-based rule with sensory and reward expectations in 80:20 (orange) and 100:0 (red) reward conditions.

B Simulated instantaneous performance plotted as a function of trial number (defined as the percentage of choices towards the option with higher pre-defined baiting probability in a 10 trial window) of an agent using a covariance-based rule with sensory expectation in 80:20 (orange) and 100:0 (red) reward conditions.

Figure S9

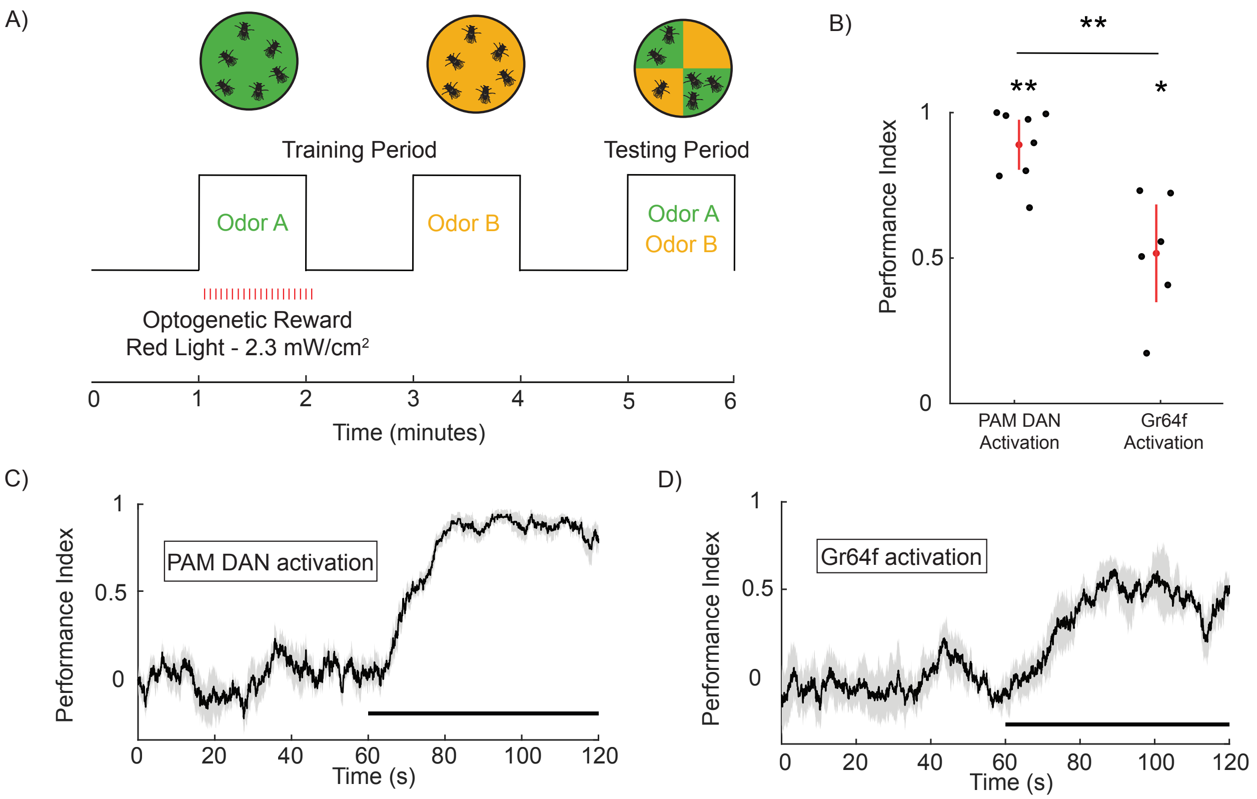

Figure S9: Circular arena experiments to control for the efficacy of optogenetic activation
A Schematic of the experimental paradigm used to train flies in the circular arena. LED intensity chosen to be 2.3 mW/cm^2 to match intensity in the Y-arena.
B Time averaged performance index (see Methods) plotted for DAN trained and Gr64f trained flies show that both learn to prefer the reward-paired odor (Wilcoxon signed-rank test: Gr64f - n = 6 groups of ~20 flies each, p = 0.0312; DAN - n = 8 groups of ~20 flies each, p = 0.0078).
C Performance index (mean in black, standard error in grey) timecourse for DAN-trained flies. The black horizontal line represents the period during which odors were present in the arena.
D Performance index (mean in black, standard error in grey) timecourse for Gr64f-trained flies. The black horizontal line represents the period during which odors were present in the arena.

Information 1: Description of the Y-arena

GENERAL NOTES:
1. MATERIAL:
2. SPECIAL FINISH: **NONE**
3. SURFACE ROUGHNESS (UNLESS SPECIFIED OTHERWISE): **N/A** (AVERAGE MICRO-INCHES)
4. INTERPRET DIMENSIONS AND TOLERANCES PER ASME Y14.5M-1994
5. DEBURR AND BREAK ALL SHARP EDGES, MAX 0.010" (UNLESS SPECIFIED OTHERWISE)
6. PARTS ARE TO BE CLEAN AND FREE OF OIL, GREASE, AND OTHER CONTAMINANTS
7. DIMENSIONS INCLUDE ANODIZING, ELECTROPLATING, AND CHEMICALLY APPLIED FINISHES IF APPLICABLE

REVISION HISTORY				
ZONE	REV	DESCRIPTION	DATE	APPROVED
	0	DO NOT FABRICATE		

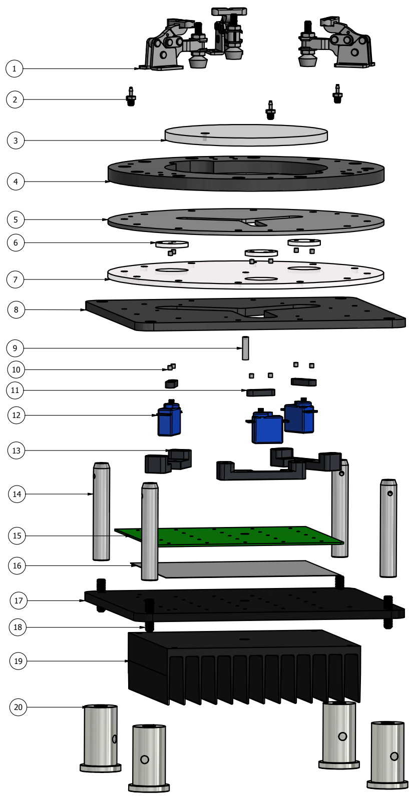

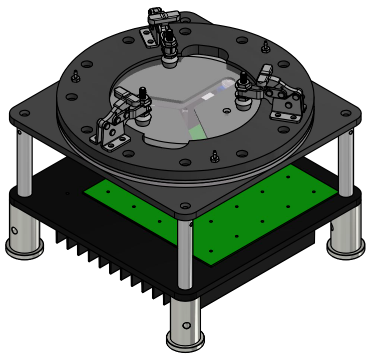

PARTS LIST

ITEM	QTY	FILE NAME	PART NUMBER	VENDOR	DESCRIPTION	MATERIAL
1	3	5004A11_Compact Hold-Down Toggle Clamp.ipt	5004A11	MCMASTER	TOGGLE CLAMPS	Generic
2	3	MCMASTER_5121K311_BARBED ADAPTER 1%16 TUBE ID.ipt	5116K115	MCMASTER	BARBED ADAPTER 1/16 TUBE ID	POLYPROPYLENE
3	1	COVER CLASS.ipt	J007739	HHMI: JRC - jET	COVER CLASS	GLASS, BOROSILICATE
4	2	CLAMPS MOUNT.ipt	J007734	HHMI: JRC - jET	CLAMPS MOUNT	ALUMINUM, 6061-T651
5	1	CHAMBER SPACER 5cm.ipt		HHMI: JRC - jET	Y CHAMBER SPACER 5CM	UHMW, WHITE
6	6	INSERT.ipt	J007738	HHMI: JRC - jET	INSERT	POLYCARBONATE, CLEAR
7	1	CHAMBER BOTTOM 5CM.ipt		HHMI: JRC - jET		POLYCARBONATE, CLEAR
8	1	CHAMBER SUPPORT 5cm.ipt	J010191	HHMI: JRC - jET	Y CHAMBER SUPPORT PLATE 5CM ARMS	ALUMINUM, 6061-T651
9	1	VACUUM FITTING.ipt	J002451	HHMI: JRC - jET	VACUUM FITTING	ACRYLIC, CLEAR
10	24	MCMASTER_5862K510_0.125 DIA 0.125 LENGTH MAGNET.ipt	5862K510	MCMASTER	0.125 DIA 0.125 LENGTH MAGNET	Generic
11	6	ARM.ipt		HHMI: JRC - jET	SERVO ARM	RESIN PRINTED VERO BLACK
12	6	HS-55_SERVO MOTOR.ipt	HS-55	SERVO CITY	SERVO MOTOR	Generic
13	6	MOTOR BRIDJE.ipt		HHMI: JRC - jET	MOTOR BRIDJE	RESIN PRINTED VERO BLACK
14	4	THORLABS_TR3_Ø0.5 IN x 3 IN OPTICAL POST.ipt	TR3	THORLABS	Ø0.5 IN X 3 IN OPTICAL POST	Generic
15	1	PCB RGB.ipt	PCB RGB	MCMASTER	PCB RGB	Generic
16	2	*Varies*	COMMON	DIGIKEY	THERMAL PAD	Generic
17	1	RGB PCB SUPPORT.ipt	J007127	HHMI: JRC - jET	RGB PCB SUPPORT	ALUMINUM, 6061-T651
18	4	MCMASTER_92313A542_CUP POINT SET SCREW 1%4-20 LENGTH 1 IN.ipt	92313A542	MCMASTER	CUP POINT SET SCREW 1/4-20 LENGTH 1 IN	STAINLESS STEEL
19	1	AAVID THERMALLOY_60140_HEAT SINK EXTRUSION.ipt	J002450	HHMI: JRC - jET	HEAT SINK EXTRUSION ALTERED	ALUMINUM, 6061-T651
20	4	THORLABS_RS2P_Ø1 IN x 2 IN OPTICAL POST.ipt	RS2P	THORLABS	Ø1 IN x 2 IN OPTICAL POST	Generic

(UNLESS SPECIFIED OTHERWISE)
PRIMARY UNITS: INCHES
[SECONDARY UNITS]: MILLIMETERS
PRIMARY TOLERANCES:

X.X	± 0.020
X.XX	± 0.010
X.XXX	± 0.005
X.XXXX	± 0.0005
ANGULAR	± 0.5 DEG

- DO NOT SCALE DRAWING -
THIRD ANGLE PROJECTION:

hhmi Howard Hughes Medical Institute — hhmi janelia Research Campus

Y ARENA.iam

SIZE	PART NUMBER	REV	SHEET
C		0	1 OF 1

Supplementary References

1. A. Dahanukar, Y.-T. Lei, J. Y. Kwon, J. R. Carlson, Two Gr genes underlie sugar reception in Drosophila. *Neuron* **56**, 503–516 (2007).
2. H. Haberkern, *et al.*, Visually Guided Behavior and Optogenetically Induced Learning in Head-Fixed Flies Exploring a Virtual Landscape. *Curr. Biol.* **29**, 1647–1659.e8 (2019).
3. N. C. Klapoetke, *et al.*, Independent optical excitation of distinct neural populations. *Nat. Methods* **11**, 338–346 (2014).
4. A. Jenett, *et al.*, A GAL4-Driver Line Resource for Drosophila Neurobiology. *Cell Rep.* **2**, 991–1001 (2012).
5. B. D. Pfeiffer, *et al.*, Refinement of tools for targeted gene expression in Drosophila. *Genetics* **186**, 735–755 (2010).
6. S. J. Gratz, *et al.*, Highly specific and efficient CRISPR/Cas9-catalyzed homology-directed repair in Drosophila. *Genetics* **196**, 961–971 (2014).
7. F. Port, S. L. Bullock, Augmenting CRISPR applications in Drosophila with tRNA-flanked sgRNAs. *Nat. Methods* **13**, 852–854 (2016).
8. Y. Aso, *et al.*, Mushroom body output neurons encode valence and guide memory-based action selection in Drosophila. *Elife* **3**, 1–42 (2014).
9. L. P. Sugrue, G. S. Corrado, W. T. Newsome, Matching behavior and the representation of value in the parietal cortex. *Science* **304**, 1782–1787 (2004).
10. B. Lau, P. W. Glimcher, Value representations in the primate striatum during matching behavior. *Neuron* **58**, 451–463 (2008).
11. K.-I. Tsutsui, F. Grabenhorst, S. Kobayashi, W. Schultz, A dynamic code for economic object valuation in prefrontal cortex neurons. *Nat. Commun.* **7**, 12554 (2016).
12. B. A. Bari, *et al.*, Stable Representations of Decision Variables for Flexible Behavior. *Neuron* **103**, 922–933 (2019)
13. B. Lau, P. W. Glimcher, Dynamic response-by-response models of matching behavior in rhesus monkeys. *J. Exp. Anal. Behav.* **84**, 555–579 (2005).
14. Y. Loewenstein, H. S. Seung, Operant matching is a generic outcome of synaptic plasticity based on the covariance between reward and neural activity. *Proc. Natl. Acad. Sci. U. S. A.* **103**, 15224–15229 (2006).
15. R. A. A. Campbell, *et al.*, Imaging a population code for odor identity in the Drosophila mushroom body. *J. Neurosci.* **33**, 10568–10581 (2013).
